# Alternative splicing and allosteric regulation modulate the chromatin binding of UHRF1

**DOI:** 10.1093/nar/gkaa520

**Published:** 2020-07-01

**Authors:** Maria Tauber, Sarah Kreuz, Alexander Lemak, Papita Mandal, Zhadyra Yerkesh, Alaguraj Veluchamy, Bothayna Al-Gashgari, Abrar Aljahani, Lorena V Cortés-Medina, Dulat Azhibek, Lixin Fan, Michelle S Ong, Shili Duan, Scott Houliston, Cheryl H Arrowsmith, Wolfgang Fischle

**Affiliations:** Laboratory of Chromatin Biochemistry, Max Planck Institute for Biophysical Chemistry, 37077 Göttingen, Germany; Biological and Environmental Science and Engineering Division, Laboratory of Chromatin Biochemistry, King Abdullah University of Science and Technology, Thuwal 23955, Saudi Arabia; Princess Margaret Cancer Centre and Department of Medical Biophysics, University of Toronto, Toronto M5G 1L7, Canada; Biological and Environmental Science and Engineering Division, Laboratory of Chromatin Biochemistry, King Abdullah University of Science and Technology, Thuwal 23955, Saudi Arabia; Biological and Environmental Science and Engineering Division, Laboratory of Chromatin Biochemistry, King Abdullah University of Science and Technology, Thuwal 23955, Saudi Arabia; Biological and Environmental Science and Engineering Division, Laboratory of Chromatin Biochemistry, King Abdullah University of Science and Technology, Thuwal 23955, Saudi Arabia; Biological and Environmental Science and Engineering Division, Laboratory of Chromatin Biochemistry, King Abdullah University of Science and Technology, Thuwal 23955, Saudi Arabia; Biological and Environmental Science and Engineering Division, Laboratory of Chromatin Biochemistry, King Abdullah University of Science and Technology, Thuwal 23955, Saudi Arabia; Biological and Environmental Science and Engineering Division, Laboratory of Chromatin Biochemistry, King Abdullah University of Science and Technology, Thuwal 23955, Saudi Arabia; Biological and Environmental Science and Engineering Division, Laboratory of Chromatin Biochemistry, King Abdullah University of Science and Technology, Thuwal 23955, Saudi Arabia; Basic Science Program, Frederick National Laboratory for Cancer Research, SAXS Core Facility of the National Cancer Institute, Frederick, MD 21702, USA; Structural Genomics Consortium, University of Toronto, Toronto M5G 1L7, Canada; Princess Margaret Cancer Centre and Department of Medical Biophysics, University of Toronto, Toronto M5G 1L7, Canada; Princess Margaret Cancer Centre and Department of Medical Biophysics, University of Toronto, Toronto M5G 1L7, Canada; Princess Margaret Cancer Centre and Department of Medical Biophysics, University of Toronto, Toronto M5G 1L7, Canada; Structural Genomics Consortium, University of Toronto, Toronto M5G 1L7, Canada; Laboratory of Chromatin Biochemistry, Max Planck Institute for Biophysical Chemistry, 37077 Göttingen, Germany; Biological and Environmental Science and Engineering Division, Laboratory of Chromatin Biochemistry, King Abdullah University of Science and Technology, Thuwal 23955, Saudi Arabia

## Abstract

UHRF1 is an important epigenetic regulator associated with apoptosis and tumour development. It is a multidomain protein that integrates readout of different histone modification states and DNA methylation with enzymatic histone ubiquitylation activity. Emerging evidence indicates that the chromatin-binding and enzymatic modules of UHRF1 do not act in isolation but interplay in a coordinated and regulated manner. Here, we compared two splicing variants (V1, V2) of murine UHRF1 (mUHRF1) with human UHRF1 (hUHRF1). We show that insertion of nine amino acids in a linker region connecting the different TTD and PHD histone modification-binding domains causes distinct H3K9me3-binding behaviour of mUHRF1 V1. Structural analysis suggests that in mUHRF1 V1, in contrast to V2 and hUHRF1, the linker is anchored in a surface groove of the TTD domain, resulting in creation of a coupled TTD-PHD module. This establishes multivalent, synergistic H3-tail binding causing distinct cellular localization and enhanced H3K9me3-nucleosome ubiquitylation activity. In contrast to hUHRF1, H3K9me3-binding of the murine proteins is not allosterically regulated by phosphatidylinositol 5-phosphate that interacts with a separate less-conserved polybasic linker region of the protein. Our results highlight the importance of flexible linkers in regulating multidomain chromatin binding proteins and point to divergent evolution of their regulation.

## INTRODUCTION

Various posttranslational modifications (PTM) of histone proteins establish binding sites for chromatin factors and serve as platforms for integrating different cellular processes (1). A number of specialized domains that recognize specific histone PTMs have been characterized. For example, chromo, chromobarrel, tudor, MBT and PWWP domains bind to histone methylation marks, bromodomain (2) and tandem PHD domains (3,4) recognize acetylation marks and SH2, BRCT, WD40 and 14–3–3 domains interact with phosphorylation marks (5). Many chromatin-binding proteins and chromatin-targeted complexes contain several domains and factors that recognize histone PTMs. The different domains either work individually/independently or in combination (bi-/multivalent or synergistic) with each other. Bi- or multivalent interactions potentially enhance overall chromatin binding. Yet, for most systems it is unclear to what degree there is synergy between individual histone PTM-binding domains (i.e. binding strength of the combined domains is more than the sum of the individual domains). Also, whether multivalent or synergistic engagement with specific modification sites on chromatin is constitutive or whether the usage of individual binding domains in composite proteins or complexes is regulated remains to be addressed.

**Figure 1.**
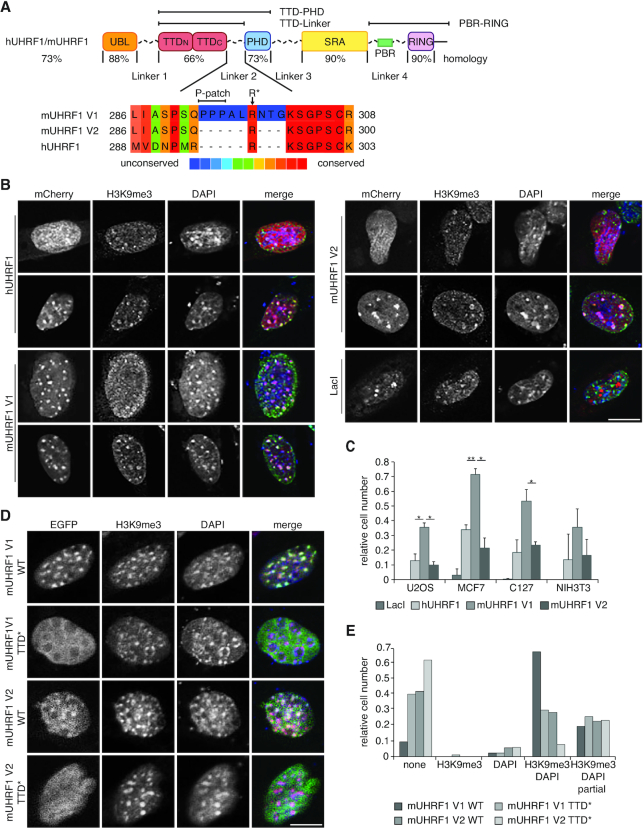
The subcellular localization of mUHRF1 V1 is different from mUHRF1 V2 and hUHRF1. (**A**) Scheme illustrating domain structure and sequence conservation of mouse and human UHRF1 (according to ClustalV). UBL, ubiquitin-like domain; TTD, tandem tudor domain (TTD_N_-TTD_C_); PHD, plant homeodomain; SRA, SET and RING associated domain; RING, really interesting new gene domain. Multiple sequence alignment (PRALINE alignment tool, http://zeus.few.vu.nl/programs/pralinewww/) of Linker 2 of the different proteins is shown. Amino acid positions correspond to the following NCBI entries: hUHRF1, NP_001276981.1; mUHRF1 V1, NP_001104550.1; mUHRF1 V2, NP_001104548.1. (**B**) Confocal images of murine C127 cells expressing mCherry-tagged murine and human UHRF1 proteins (mCherry, red channel). Cell populations showed different distribution of UHRF1. Representative cells of diffuse (top row) and focal (bottom row) UHRF1 nuclear distribution are shown. Immunofluorescence staining was performed for H3K9me3 (green channel). DAPI staining marks the DNA (blue channel). Merged images show all three channels simultaneously. Scale bar: 15 μm. (**C**) Co-localization of H3K9me3 and mCherry-tagged proteins as shown in (B) was assessed visually. Data are presented as mean and standard deviation (s.d.) of three independent experiments (*n* > 100). Unpaired two-sided student's t-test was performed to compare samples; (*) *P* < 0.05, (**) *P* < 0.01. (**D**) Representative confocal images of murine NIH-3T3 cells expressing EGFP-mUHRF1 V1 and V2 WT and Y184/Y187A (TTD*) mutant proteins (EGFP, green channel). Immunofluorescence staining was performed for H3K9me3 (red channel). DAPI staining marks the DNA (blue channel). Merged images show all three channels simultaneously. Scale bar: 10 μm. (**E**) Co-localization of EGFP-tagged proteins as shown in (D) with H3K9me3 and DAPI-dense regions was assessed visually and is plotted relative to the total number of EGFP-positive cells (*n* > 70).

Ubiquitin-like with PHD and RING Finger domains 1 (UHRF1), also known as Nuclear Protein of 95 kDa (NP95) in mouse, is a multi-domain nuclear factor containing a combination of three domains recognizing different chromatin marks paired with enzymatic E3 ubiquitin ligase activity. From N- to C-terminus the protein is composed of a ubiquitin-like domain (UBL), a tandem tudor domain (TTD), a plant homeodomain (PHD), a SET and RING-associated (SRA) domain, and a ‘really interesting new gene’ (RING) domain. The functionally and structurally defined domains of UHRF1 are connected by linker regions of various lengths (Figure 1A). The TTD recognizes the H3K9me3 mark (6,7) and K126me of DNA ligase 1 (LIG1) (8). The PHD interacts with the unmodified N-terminus of H3 (9), while the SRA domain binds hemi-methylated DNA (10,11). The RING domain has E3 ubiquitin ligase activity for histone H3 residues K14, 18 and/or 23 (12,13). Recently, the UBL was shown to be essential for H3 ubiquitylation in a nucleosomal context (14).

UHRF1 is involved in regulation of the cell cycle and replication-dependent DNA damage control (15–19). Deletion of UHRF1 in mice results in severe loss of global DNA methylation and embryonic lethality after gastrulation (20). Indeed, UHRF1 has emerged as an essential protein for DNA maintenance methylation (20,21). The protein is thought to recognize hemimethylated DNA in chromatin context, an event that triggers its H3 ubiquitylation activity (22,23). Ubiquitylated H3 in turn has been implied in recruiting the maintenance methyltransferase DNMT1 to nascent chromatin (24–26).

Based on other and our work, we have proposed before that the multivalent interaction of UHRF1 with histone and DNA modifications is dictated by linker-mediated inter-domain communication within UHRF1 (27). For instance, we have shown that UHRF1 histone-binding is allosterically regulated by the phosphatidylinositol phosphate PI5P. This ligand binds to a polybasic region (PBR) of Linker 4. In the apo state the PBR is bound to the TTD thereby blocking its interaction with the H3-tail. PI5P-binding releases the PBR from the TTD, which in turn enables recognition of H3K9me3 (28).

The sequences and structural details of the different domains of UHRF1 are highly conserved (29) (Figure 1A). It is, therefore, mostly assumed that UHRF1 proteins of different origin share a common mode of action. As a result, different studies have used UHRF1 proteins from different species (e.g. *Homo sapiens*, *Mus musculus, Danio rerio* and *Xenopus laevis*) interchangeably and made generic interpretations about the function of the protein (6,12,14,21,30,31). Yet, the sequence variability of the flexible linkers in different species has until now not been taken into consideration. In this study, we identified two murine UHRF1 splicing variants, which only differ in the Linker 2 between the TTD and PHD by an insertion of nine amino acids (Figure 1A). This results in altered subnuclear localization, recruitment to H3K9me3 and H3 ubiquitylation activity of the protein. Using functional and structural approaches, we elucidated the molecular mechanisms that give rise to different functional outcomes of mouse variants and human UHRF1. Our results highlight the importance of flexible linkers in regulating multidomain chromatin-binding proteins and point to divergent evolution of their regulation.

## MATERIALS AND METHODS

### mRNA expression analysis

Mouse mm10 transcriptome data (Poly-A plus RNAseq) for available tissue categories and different embryo stages were obtained from http://genome.crg.es/encode_RNA_dashboard. Reads were mapped to genome version mm10 and quantified using STAR aligner (32) and HTseq (33). Four different mUHRF1 transcript annotations from GencodeM24 were used for quantification: mUHRF1 V1 is encoded by transcripts Uhrf1–201 (ENSMUST00000001258.10) and Uhrf1–204 (ENSMUST00000113039.4), mUHRF1 V2 is encoded by transcripts Uhrf1–202 (ENSMUST00000113035.3) and Uhrf1–203 (ENSMUST00000113038.3). Proportion of transcripts (TPM – transcripts per million) was estimated from the HTseq read count. Visualization of quantified transcript variants was performed using clustergrammer (34).

### Recombinant proteins

cDNAs representing full-length and individual domains of hUHRF1, mUHRF1 V1 and V2 were cloned into the petM13 vector (EMBL) for expression of 6× His-tag and/ or Myc-tag fusion proteins. Proteins were expressed in BL21(DE3) RIL bacteria growing in 2× YT media, purified using HisPur Cobalt-Resin (Thermo-Fisher-Scientific) and dialyzed to storage buffer (50 mM Tris–HCl pH 7.5, 150 mM NaCl, 10% (v/v) glycerol, 1 mM DTT). After concentration using Amicon Ultra centrifugation filter units, proteins were stored at 4°C or –20°C. Further details are available upon request.

### Peptides

The following peptide backbones were obtained from Synpeptide Co., Ltd: H3 aa 1–15 (ARTKQTARKSTGGKA) and H3 aa 1–20 (ARTKQTARKSTGGKAPRKQL). Modifications (K9 trimethylated or R2 symmetrically dimethylated and K9 trimethylated) and functionalization (labelling with biotin or fluorescein) were incorporated at synthesis. mPBR corresponds to the sequence SKTGKSKQKSTGPTLS and hPBR to GKGKWKRKSAGGGPS.

### Cell culture and transfection

Tissue culture cells were maintained at 37°C, 5% CO_2_ in DMEM media supplemented with 10% (v/v) FCS and 1% (v/v) MEM-NEAA with the following additions: U2OS (human osteosarcoma), NIH-3T3 (murine fibroblast) with 1 g/l glucose and 1× GlutaMAX; MCF7 (human breast cancer) with 1 g/l glucose and 2× GlutaMAX; and C127 (mouse mammary gland) cells with 4.5 g/l glucose and 1× GlutaMAX. For transfection of human and murine cell lines, cDNAs corresponding to full-length hUHRF1, mUHRF1 V1 and V2 were cloned into pmCherry-C1 or pEGFP-N2 vectors (Clontech) for expression with mCherry- or EGFP-tags, respectively. Cells were seeded into 10 cm dishes for lysate preparation or on coverslips for immunofluorescence. U2OS, MCF7 and NIH-3T3 cells were transfected using Lipofectamine LTX Kit (Thermo-Fisher-Scientific). C127 cells were electroporated using the Neon electroporation system (ThermoFisher Scientific) at 1400 V, with two pulses of 20 ms.

### Immunofluorescence

Cells were fixed with 4% (v/v) formaldehyde in cell culture media (10% FCS supplemented) and permeabilized three times 5 min with 0.1% Triton X-100 (v/v) in PBS. Cells were then incubated with primary antibody (rabbit anti-H3K9me3, Active Motif 39161, 1:500) in PBS, 4% (v/v) FCS overnight. After washing three times 10 min in PBS, secondary antibody (anti-rabbit Alexa Flour^®^ 488, A21206, or anti-rabbit Alexa Flour^®^ 568, A10042, Thermo Fisher, 1:500) was added in PBS, 4% (v/v) FCS for 1.5 h. DNA was stained with 1 μg/ml DAPI for 2 min and slides were mounted with 40 μl ProLong^®^ Diamond anti-fade mountant. Immunofluorescence analysis was performed blinded on Zeiss LSM 710 or Leica SP6 microscopes at 63× magnification. For Fiji intensity plots three-colour merged images were generated in Adobe Photoshop. Linear image manipulation was done where necessary. Fiji was used to mark a region of interest, for which a three-colour intensity plot was computed.

### Flow cytometry

Cells were trypsinized and resuspended in PBS for analysis on a BD FACSCanto™. Cell populations were gated based on forward and side scatter. mCherry or EGFP fluorescence were recorded in the PI channel (ex 488 nm, em 610/10) or in the GFP channel (ex 488 nm, em 530/30), respectively. Post-recording analysis was performed using FloJo software.

### Nuclear extracts

Cells in 10 cm dishes were transfected at 50% confluency. At 80% confluency cells were washed once with cold PBS, before being scraped, pelleted and lysed in 100 μl PBS, 0.1% (v/v) NP40 by pipetting. Nuclei were recovered by short centrifugation and the supernatant was discarded. After washing once with ice-cold PBS, nuclei were lysed successively in 100 μl strip buffer (10 mM Tris–HCl pH 7.4, 1 mM EGTA, 1.5 mM KCl, 5 mM MgCl_2_, 290 mM sucrose, 0.1% (v/v) Triton X-100, 1 mM DTT, 1× cOmplete mini EDTA-free (Roche)), 100 μl low/medium/high salt lysis buffer (20 mM HEPES–KOH pH 7.9, 25% glycerol, 200/400/800 mM KCl, 1.5 mM MgCl_2_, 0.2 mM EDTA, 1 mM DTT, 1× cOmplete mini EDTA-free (Roche)) for 5 min on ice each. Supernatants were combined and salt concentration was adjusted to 150 mM KCl with lysis buffer without KCl. Extract was cleared at 21 000 × g for 5 min and directly used for pull-down experiments.

### Peptide pull-down

40 μl streptavidin paramagnetic beads (Promega) per pull-down were washed three times with PD150 buffer (20 mM HEPES–KOH pH 7.9, 150 mM KCl, 0.2% (v/v) Triton X-100, 20% (v/v) glycerol). 10 μg synthetic, biotin-labelled H3 aa 1–20 peptides were added in PD150 buffer for 1 h at RT with rotation. Beads were washed three times with PD150 buffer before adding 200 μl nuclear extract for 3 h at 4°C with rotation. Beads were washed three times 5 min with PD150; all supernatant was discarded, and the beads were eluted in 20 μl 1.5× SDS loading buffer (94 mM Tris–HCl pH 6.8, 3% (w/v) SDS, 6.5% (v/v) glycerol, 150 μg/ ml bromophenol blue, 15 mM TCEP).

### Co-immunoprecipitation

10 μl Myc-tag mouse mAB magnetic bead conjugate (Pierce) were washed once with PBS and twice with IP150 buffer (50 mM Tris–HCl, pH 8, 150 mM NaCl, 5% (v/v) glycerol, 0.05% (v/v) Triton X-100). 0.44 nmol recombinant m/hUHRF1 Myc-PBR-RING was added for 3 h at 4°C with rotation in 100 μl IP150 buffer, 5% (w/v) BSA. Reactions were supplemented with 0.44 nmol of recombinant 6× His tagged TTD-PHD of hUHFR1, mUHRF1 V1 or V2 in 100 μl IP150 buffer, 5% (w/v) BSA. After 3 h incubation, reactions were washed four times 1 min in 150 μl IP150 buffer and eluted in 20 μl 1.5× SDS loading buffer.

### Western blotting

Samples in SDS loading buffer were boiled for 5 min and run on 10% or 15% polyacrylamide gels. After transfer to nitrocellulose or PVDF, membranes were blocked in PBST, 5% (w/v) dry milk powder or 2% (w/v) BSA. Primary antibodies were added in blocking buffer overnight: rabbit anti-mCherry (ThermoFisher, PA5–34974, 1:10 000); mouse anti-HP1β (Millipore, MAB-3448, 1:1000); mouse anti-GFP (Santa Cruz, sc-9996, 1:1000 to 1:5000); mouse anti-UHRF1 (Santa Cruz, sc-373750, 1:1000); mouse anti-His-tag (Santa Cruz, sc-57598, 1:500); mouse anti-FLAG (Sigma, F1804, 1:2000). Membranes were washed three times 10 min with PBST and incubated with secondary antibodies (anti-rabbit HRP, Dako, P0399, 1:10 000; anti-mouse HRP, Dako, P0447, 1:10 000 or anti-mouse IRDye 800CW, LI-COR Biosciences, 926-32210, 1:10 000) in blocking buffer for 1 h at room temperature. Membranes were washed three times 10 min with PBST and incubated with ECL substrate for 2 min before imaging on a ChemiDoc (Bio-Rad) or dried and imaged on a LI-COR Odyssey^®^ CLx.

### Quantitative binding measurements

Fluorescence polarization (FP) analysis was carried out as described (28) on HIDEX PlateChameleon or TECAN Infinite M1000 Pro plate readers at RT.

For microscale thermophoresis (MST), 6× His-tagged proteins were labelled using Monolith His-tag labelling kit RED-tris-NTA (NanoTemper; MO-L008). 400 nM protein was incubated with 100 nM His-tag labelling dye in MST buffer (20 mM HEPES–NaOH pH 7.9, 150 mM NaCl, 0.05% (v/v) Tween-20) for 30 min at room temperature, followed by centrifugation at 15 000 × g for 10 min at 4°C. Titration series of 50 nM fluorophore-labelled protein with peptides or 16:0 phosphatidylinositol 5-phosphate (Echelon P5016) were incubated at room temperature for 15 min before measuring on Monolith NT.115 (NanoTemper, 80% LED power, 40% MST power). Data points were fitted using the following equation:

$$\begin{eqnarray*}\left[ {{\rm{AL}}} \right]{\rm{ = \raise.5ex\hbox{$\scriptstyle 1$}\kern-.1em/ \kern-.15em\lower.25ex\hbox{$\scriptstyle 2$} *}}\left( {\left( {\left[ {{{\rm{A}}_{\rm{0}}}} \right]{\rm{ + }}\left[ {{{\rm{L}}_{\rm{0}}}} \right]{\rm{ + }}{{\rm{K}}_{\rm{D}}}} \right){\rm{ - }}{{\left( {{{\left( {\left[ {{{\rm{A}}_{\rm{0}}}} \right]{\rm{ + }}\left[ {{{\rm{L}}_{\rm{0}}}} \right]{\rm{ + }}{{\rm{K}}_{\rm{D}}}} \right)}^{\rm{2}}}{\rm{ - 4*}}\left[ {{{\rm{A}}_{\rm{0}}}} \right]{\rm{*}}\left[ {{{\rm{L}}_{\rm{0}}}} \right]} \right)}^{{\rm{1/2}}}}} \right)\end{eqnarray*}$$

*K*_D_, dissociation constant; [A_0_], concentration of fluorescent molecule; [L_0_], concentration of ligand/binding partner; [AL], concentration of the complex of A and L.

All FP and MST binding measurements were performed as biological and technical replicates with at least two independent protein preparations. Raw fluorescent values of each data set were normalized using the following formula:

$$\begin{equation*}{{y = }}\left( {{{{y}}_{\rm{0}}}{\rm{- min}}} \right){\rm{/}}\left( {{\rm{max - min}}} \right)\end{equation*}$$

where max and min values were defined from the curve fitting to a single binding site model. Normalized data were averaged and plotted using GraphPad Prism.

### Nucleosome reconstitution

Nucleosomes were reconstituted as described (35), using recombinant *H. sapiens* core histones and 187 bp DNA fragments containing the 601 DNA sequence at its centre (36). H3K9me3 was generated by native chemical ligation as described (37).

### Ubiquitylation assays

Reactions were performed in 20 μL volume containing 0.5 μM UHRF1, 100 nM E1 activating enzyme, 200 nM E2 UbcH5b (Boston Biochem), 5 mM Mg-ATP, 5 μM FLAG-ubiquitin (Boston Biochem), 20 mM HEPES–NaOH pH 7.9, 100 mM NaCl, and 1 μM nucleosomes at 24°C. Incubations were stopped by adding SDS-PAGE loading buffer and boiling.

### NMR

NMR spectra were acquired on Bruker spectrometers operating at 500, 600 or 800 MHz, equipped with TCI cryoprobes in 20 mM sodium phosphate pH 7.5, 150 mM NaCl, 5 mM DTT, 5 mM beta-mercaptoethanol, 2 mM TCEP and 10 μM ZnSO_4_. ^1^H, ^13^C and ^15^N resonance assignments were determined for ^15^N/^13^C-labelled hTTD-Linker 2 (hUHRF1_126–301_, 88% complete) and mTTD-Linker 2 V1 (mUHRF11_122–304_, 85% complete) and mPHD (mUHRF1 V1_303–380_, 96% complete) using FMCGUI (38) based on the following 3D triple and double-resonance NMR experiments: HNCO, CBCA(CO)NH, HBHA(CO)NH, HNCA, (H)CCH-TOCSY and H(C)CH-TOCOSY, ^15^N-edited NOESY-HSQC and ^13^C-edited NOESY-HSQC. Aromatic ring resonances were assigned using a 3D ^13^C-edited NOESY spectrum with carbon pulses centred at 122 ppm. All 3D spectra were acquired with non-uniform sampling in the indirect dimensions and were reconstructed by multi-dimensional decomposition software MDD-NMR (39) or qMDD (40), interfaced with NMRPipe (41).

Distance restraints for structure calculations were derived from cross-peaks in the NOESY-HSQC spectra. Peak picking was performed manually using Sparky (T.D. Goddard and D.G. Kneller, SPARKY 3, University of California, San Francisco). Torsion angle restraints were derived from TALOS+ (42). Hydrogen bond restraints were applied only for residues that were clearly in secondary structure regions as judged by NOE patterns, and chemical shifts supported by TALOS+. Automated NOE assignments and structure calculations were performed using CYANA 2.1 (43). The best 20 of 100 CYANA-calculated structures were refined with CNSSOLVE (44) by performing a short restrained molecular dynamics simulation in explicit solvent (45). The final 20 refined structures compose the NMR ensemble. The quality of the NMR structures was assessed by PSVS (46). For mPHD, to find out which residues are coordinating the three Zn ions, the initial structural ensemble was determined using only NOE distance and dihedral angle restraints, without including the Zn ions in the calculation. The Zn1 ion is coordinated by four Cys residues (Cys307, Cys310, Cys318 and Cys321), the Zn2 ion is coordinated by three Cys and one His residues (Cys323, Cys326, His346 and Cys349), and the Zn3 ion is coordinated by four Cys residues (Cys338, Cys341, Cys365 and Cys368). Both the Cα and Cβ chemical shifts for all eleven coordinating Cys residues were in good agreement with what is expected for Cys ligated to a zinc ion (47). The position of the His346 side chain was well defined by 13 experimental NOE distance restrains, and they clearly indicated that Nδ1 (not Nϵ2) is ligated to the Zn2 ion. Additional non-experimental distance restraints involving zinc and its coordinating residues were introduced in the CYANA calculation for the final ensemble. In order to maintain proper tetrahedral geometry around coordinated Zn, the distances between pairs of atoms (i.e. Zn-Cys S_γ_, Zn-Cys C_β_, His N_δ1_-Zn, His N_δ1_-Cys S_γ_ and Cys S_γ_-Cys S_γ_) were restrained as previously described (48). These restraints were sufficient to keep the zinc coordination geometry intact.

### SAXS and molecular modelling

Samples were measured in 20 mM HEPES–NaOH pH 7.5, 150 mM NaCl, 10 μM ZnCl_2_, 0.5 mM TCEP and 2 mM DTT at the beamline 12-ID-C of the Advanced Photon Source, Argonne National Laboratory (14.0 keV, wavelength λ = 0.8856 Å, sample to detector distance = 1.9 m to achieve a *q* range of 0.0045 < *q* < 0.990 Å^–1^, where *q* = (4π/λ)sin θ, and 2θ is the scattering angle). Thirty 2D SAXS intensity maps were recorded with a Pilatus 2 M pixel detector with an exposure time of 0.5–1.0 s. Data were analysed with ATSAS 2 (49). The experimental radius of gyration, *R*_g,_ was calculated from data at low *q* values using the Guinier approximation. The pair distance distribution function (PDDF), p(r), and the maximum dimension of the protein, *D*_max_, in real space was calculated with the indirect Fourier transform using GNOM (50). Estimation of the molecular weight of samples was obtained by both SAXSMOW (51,52) and by using the Volume of correlation, *V*_c_ (53). The theoretical scattering intensity of the atomic structure model was fitted to the experimental scattering intensity using FoXS (54).

We used SAXS data in combination with rigid-body modelling to test the conformational flexibility of TTD-PHD modules. *R*_g_ distributions were calculated by SAXS-driven ensemble fitting using SES method (55). Initial ensemble consisting of 30 000 possible relative configurations of TTD and PHD domains were generated by RANCH (56,57). In the simulations, Linker 2 between the PHD and TTD (residues 279–306 and 279–298 for mUHRF1 V1 and V2, respectively) were assumed to be flexible, so that the initial ensemble of conformations represents all possible random configurations of the TTD-PHD modules.

## RESULTS

### A splicing variant of mUHRF1 shows altered subnuclear localization and recruitment to H3K9me3

Previous studies reported conflicting results regarding the mechanisms of UHRF1 recruitment to chromatin and the requirement of different UHRF1 domains for DNA maintenance methylation. For example, while several studies show that both functional TTD and/or PHD domains are required for chromatin binding, focal nuclear localization, and the DNA maintenance methylation function of human and mouse UHRF1 (7,28,30), it is still under debate which chromatin ligand is actually involved in UHRF1 recruitment: H3K9me2/3, TOP2A, LIG1 or others (7,8,31,58). Moreover, results obtained from other studies suggest that the SRA domain and hemimethylated DNA are the primary determinants for UHRF1 chromatin localization and DNA maintenance methylation (12,20,59–61). Another report implied that DNA binding by the SRA domain is important for chromatin localization, but its ability to specifically recognize hemimethylated DNA is not (22). Two recent studies found that a functional TTD is not required for DNA methylation maintenance in mice (61) or human cancer cells (62). These conflicting results might be partially due to murine UHRF1 (mUHRF1) and human UHRF1 (hUHRF1) proteins being regarded as interchangeable and being studied in various cell systems where species were not always matched (7,12,20,28,30,31,59). We uncovered that, in mouse, alternative splicing of exon eight gives rise to two different variants of mUHRF1 only differing in the mLinker 2 region between the mTTD and mPHD histone modification reader domains. According to NCBI nomenclature, we refer to mUHRF1 V1 as the variant that has, to our knowledge, been used by all previous studies. mLinker 2 V1 has an insertion of nine amino acids in its centre compared to variant 2 (V2). mLinker 2 V2 is, in contrast, highly similar to hLinker 2 (Figure 1A).

In the absence of antibodies that could distinguish the two mUHRF1 proteins, we analysed RNA-seq data from different mouse developmental stages as well as different adult tissues (8 weeks old mice) to determine expression of four mRNA species corresponding to the two protein variants. Both variants are expressed as two mRNAs each that differ in their 5′UTR (mUHRF1 V1: Uhrf1–201 and -204, mUHRF1 V2: Uhrf1–202 and 203). We found that mUHRF1 V1 is the predominantly expressed isoform, both during embryonic development and in adult tissues (Supplementary Figure S1). As expected from its role in maintenance DNA methylation, mUHRF1 expression is highest in tissues with actively and rapidly dividing cells: the intestine, immune system compartments (thymus, bone marrow, spleen), as well as the gonads and reproductive organs (mammary gland, placenta). The stomach and duodenum are the only organs displaying higher expression of mUHRF1 V2 compared to V1. Terminally differentiated cells (brain, muscle cells, kidney, liver) show low to undetectable mRNA levels for both mUHRF1 variants. Consistently, all early embryonic developing tissues (E11.5, E14) express high levels of mUHRF1, which decreases with maturation of the embryo, and its organs (E14, E14.5, E18) (Supplementary Figure S1).

Because of the observed differences in the expression profiles of mUHRF1 splicing variants, we reasoned that they might have distinct biological properties. We first analysed the subnuclear localization of the two murine UHRF1 variants and the human protein, hypothesizing that this might reveal divergent functionalities. We transiently expressed mCherry-tagged hUHRF1, mUHRF1 V1, V2 or LacI in different human as well as murine cell lines. Co-localization of the mCherry-tagged proteins with DAPI-dense regions and H3K9me3 in the nucleus of these cells was examined using confocal microscopy and subsequent analysis via Fiji intensity plots (Figure 1B, Supplementary Figure S2A). To exclude effects of differing expression on the observed variations in subnuclear distribution, we verified comparable protein concentrations on the level of cell culture ensembles by western blotting (Supplementary Figure S2B) and in individual cells using flow cytometric analysis (Supplementary Figures S3 and S4). Regardless of the differences in transfection efficiency and global expression levels of the three proteins in different cell systems, we found a reproducible pattern in all investigated cell lines (Figure 1B, C). In human U2OS osteosarcoma, human MCF7 breast cancer, mouse C127 mammary gland and mouse NIH-3T3 fibroblast cells a significantly smaller fraction showed co-localization of mCherry-hUHRF1 (13%, 34%, 19% and 13%, respectively) and mCherry-mUHRF1 V2 (10%, 21%, 23% and 17%, respectively) with H3K9me3 when compared to mCherry-mUHRF1 V1 (35%, 71%, 53% and 36%, respectively). Based on these observations, we concluded that the chromatin association of the two murine and single human UHRF1 proteins are distinct.

Previous studies suggested that the connected TTD and PHD (TTD-PHD module) regulate subnuclear localization and UHRF1 ubiquitylation activity. However, the importance of the TTD domain in chromatin targeting of UHRF1 is still under debate (7,59,61,62). Since we found significant differences in subnuclear localization in respect to H3K9me3, we mutated the aromatic cage in the mTTD domain (mTTD*: Y184A/Y187A) and overexpressed EGFP-mUHRF1 V1 and V2 WT and TTD* in C127 and NIH-3T3 cells (Figure 1D, Supplementary Figure S5A). Using confocal microscopy, we analysed the co-localization of EGFP with H3K9me3 and found that mutation of the mTTD decreased mUHRF1 V1 co-localization with H3K9me3 to the level of mUHRF1 V2 (Figure 1E, Supplementary Figure S5B). mUHRF1 V2 TTD* displayed even lower co-localization with H3K9me3 in both cell lines. These results indicated that a functional mTTD domain is essential for mUHRF1 subnuclear localization.

### H3K9me3 recognition of mUHRF1 variants is dependent on distinct behaviour of the mTTD

**Figure 2.**
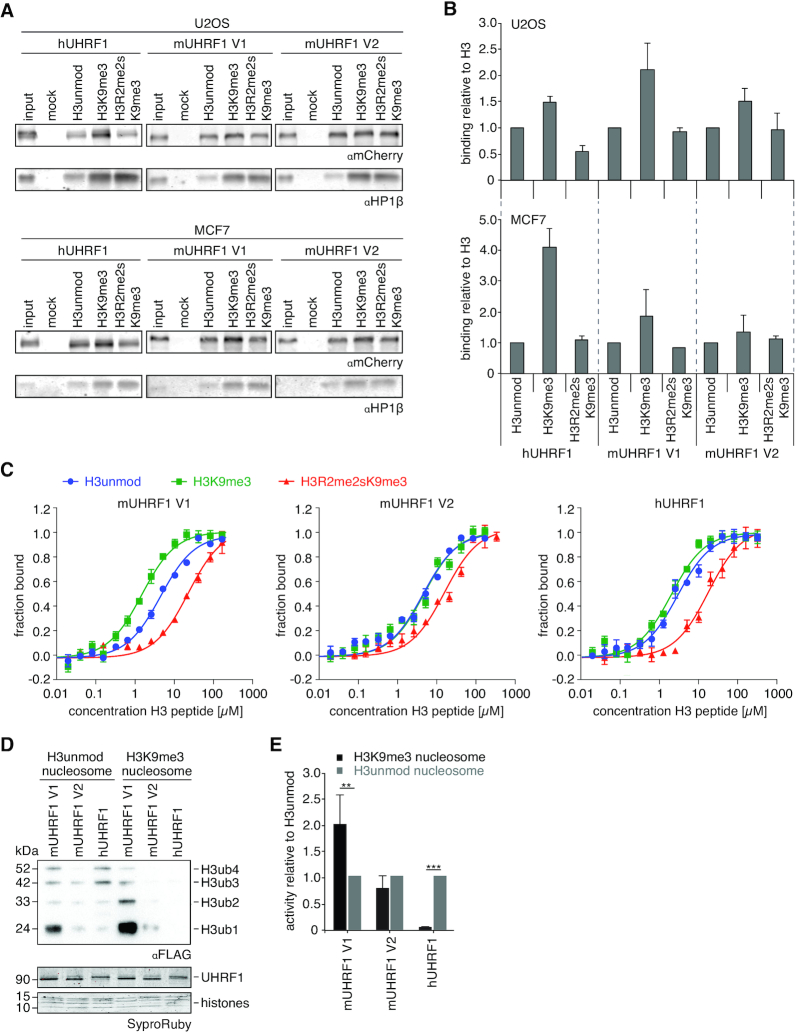
H3K9me3 recognition is dependent on the availability of the TTD. (**A**) Representative histone-peptide pull-down experiment using extracts from mCherry-hUHRF1 or -mUHRF1 expressing U2OS and MCF7 cells. Material recovered on immobilized peptides was analysed by western blot. HP1β serves as control for a *bona fide* H3K9me3-binding protein. (**B**) Quantification of anti-mCherry western blot signals corresponding to the experiments shown in (A) relative to immobilized peptide. Data are presented as mean and s.d. of two (H3R2me2sK9me3) or three (H3 unmodified, H3K9me3) independent experiments. (**C**) Titration series of different H3 peptides with fluorescently labelled, recombinant UHRF1 proteins were analysed by microscale thermophoresis. Data are plotted as average of three independent experiments; error bars correspond to s.d. (**D**) Recombinant UHRF1 proteins were subjected to E3 ubiquitin ligase assays using recombinant unmodified or H3K9me3 mononucleosomes. Reactions were analysed by western blotting (top). Signals for UHRF1 and histone proteins on the stained western blot membrane are shown for loading controls (bottom). Running positions of molecular weight markers (left) and different identified proteins (right) are indicated. (**E**) Quantification of anti-FLAG western blot signals obtained for ubiquitination assays as shown in (D). Absolute values were quantified using ImageJ and normalized to the H3unmod nucleosome signal. Data are presented as mean and s.d. of four (mUHRF1 V1, hUHRF1) and two (mUHRF1 V2) independent experiments. Student's *t*-test (unpaired, two-tailed) was performed to compare samples; (**) *P* < 0.01, (***) *P* < 0.001.

To gain further insights into the mechanism behind the functional differences between mUHRF1 V1, V2 and hUHRF1, we performed histone-peptide pull down experiments using cell lysates from mCherry-mUHRF1 V1, V2 and -hUHRF1 expressing cells (U2OS and MCF7). We compared the recovery of UHRF1 proteins on three differently modified peptides corresponding to residues 1–20 of the H3-tail. Based on other (6,7,9,61,63–66) and our (28) previous observations, we rationalized that the H3 unmodified peptide recovers only PHD-dependent interaction, H3K9me3 binds to TTD and PHD either independently, or bivalently with or without synergism, and H3R2me2sK9me3 impedes, but does not fully block, PHD-binding, while allowing for TTD-dependent methylation-specific peptide recognition. We found hUHRF1 and mUHRF1 V1 preferentially enriched on the H3K9me3 peptide. In contrast, mUHRF1 V2 showed lower preference for this peptide over H3 unmodified and H3R2me2sK9me3 (Figure 2A, B).

We have previously shown that hUHRF1 exists in different H3K9me3-binding states in native (i.e. cell lysate) versus recombinant systems (28). To assess whether similar differences exist for the murine variants, we compared the peptide binding behaviour of recombinant proteins in quantitative microscale thermophoresis (MST) experiments. Unlike hUHRF1, recombinant mUHRF1 V1 and V2 proteins showed similar peptide binding pattern when compared with the cellular proteins. Mouse V1 exhibited significant binding preference for H3K9me3 over the unmodified counterpart (Figure 2C) suggesting recognition by the mTTD (see Supplementary Table S1 for a listing of all *K*_D_ values measured in this study). Further, it showed 20-fold reduced binding to the H3R2me2sK9me3 peptide indicating that mUHRF1 V1 establishes a bivalent and synergistic binding mode where both mTTD and mPHD domains engage with the target. In contrast, mUHRF1 V2 had no binding preference for the H3K9me3 over the unmodified peptide. It also showed far less reduction in interaction with the H3R2me2sK9me3 compared to the H3K9me3 peptide (Figure 2C). This suggested that mTTD and mPHD domains function independent of each other in mUHRF1 V2. In these assays, hUHRF1 showed no significant preference for the H3K9me3 mark over unmodified and 10-fold weaker affinity to the doubly modified peptide (H3R2me2sK9me3) compared to H3K9me3, confirming a hPHD-dominated binding mode.

The distinct binding patterns of the three recombinant proteins were confirmed in an independent assay system using N- and C-terminally labelled fluorescent H3 peptides (fluorescence polarization, FP; Supplementary Figure S6A). In addition, we compared E3 ligase activity of murine and human UHRF1 in an *in vitro* ubiquitylation assay using nucleosomal substrates containing either unmodified or K9me3 H3. mUHRF1 V1 was more active in ubiquitylating H3 in the context of unmodified nucleosomes compared to V2 and hUHRF1 (Figure 2D). This activity of V1 was further enhanced on H3K9me3 nucleosomes, whereas V2 and hUHRF1 showed no such stimulation (Figure 2D, E). Overall, our results from the binding experiments with cellular and recombinant proteins as well as the ubiquitylation assay indicate that, due to different functional states of the TTD domains, the two mUHRF1 V1 and V2 variant proteins differ from each other, as well as from hUHRF1 with respect to their H3K9me3 recognition.

### Different molecular mechanisms determine the functional state of the TTD in murine and human UHRF1

**Figure 3.**
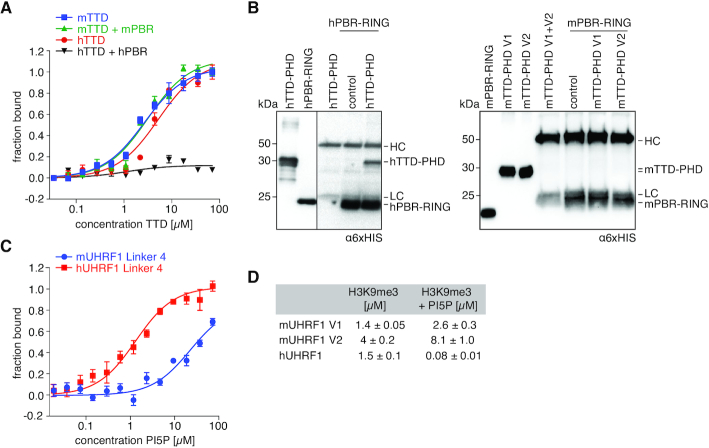
mTTD is not regulated by mLinker 4/ mPBR. (**A**) Titration series of recombinant mTTD and hTTD with a FAM-H3(1–15)K9me3 peptide with and without twofold molar excess of oligopeptides corresponding to mPBR or hPBR were analysed by fluorescence polarization. Data are plotted as average of three independent experiments; error bars correspond to s.d. (**B**) Recombinant mPBR-RING and hPBR-RING proteins were immobilized on magnetic beads and incubated with mTTD-PHD V1, V2 or hTTD-PHD. Protein input and recovered material were analysed by western blot. Running positions of molecular weight markers (left) and different identified proteins (right) are indicated. (**C**) Titration series of PI5P with fluorescently labelled, recombinant mLinker 4 or hLinker 4 was analysed by microscale thermophoresis. Data are plotted as average of three independent experiments; error bars correspond to s.d. (**D**) Dissociation constants (*K*_D_) for H3K9me3 peptide binding in the absence and presence of 100-fold molar excess of 16:0 PI5P over recombinant murine and human UHRF1 proteins as determined by microscale thermophoresis. Results represent averages of minimally three independent experiments; error bars correspond to s.d.

In hUHRF1, a polybasic region (hPBR) of hLinker 4 regulates the functional state of the hTTD by blocking its peptide-binding surface groove (28,67). Sequence comparison revealed conservation of a basic patch in the PBR of human and murine UHRF1 proteins (Supplementary Figure S6B). We compared H3K9me3-binding of mTTD and hTTD domains in the absence and presence of corresponding PBR peptides. Isolated TTDs from both species showed similar binding to the H3K9me3 peptide (Figure 3A, Supplementary Figure S6C). As previously described, hPBR blocked the binding of hTTD to H3K9me3. In contrast, mPBR did not have any effect on mTTD/H3K9me3 interaction (Figure 3A, Supplementary Figure S6D).

To evaluate direct interaction between the TTD-PHDs and the C-terminal regions of the different UHRF1 proteins, we performed immunoprecipitation experiments. We found that, regardless of the sequence similarities, the strong interaction of the PBR-RING with the TTD-PHD is only present in hUHRF1 but not in mUHRF1 V1 and V2 (Figure 3B). These results are consistent with the human R649 residue, which has been shown to occupy the R-pocket in the hTTD surface groove (67), not being conserved in the mPBR sequence (Supplementary Figure S6B).

We have shown before that phosphatidylinositol 5-phosphate (PI5P) unblocks the hTTD by interacting with the hPBR. This enables H3K9me3 recognition of hUHRF1 (28). To test whether a similar regulatory mechanism exists in mUHRF1 V1 and V2, we measured PI5P binding to the PBR-containing Linker 4 of the different proteins using MST. hLinker 4 bound PI5P with a *K*_D_ of 1.4 μM (Figure 3C). Addition of PI5P significantly enhanced H3K9me3-binding of hUHRF1, indicating unblocking of the hTTD concomitant with synergism of the hTTD-PHD module (Figure 3D). Compared to the human protein, mLinker 4 showed only weak affinity for PI5P (*K*_D_ > 80 μM, Figure 3C). Also, the phospholipid did not have any effect on H3K9me3 mark recognition by mUHRF1 V1 and V2 (Figure 3D). Further and in contrast to hUHRF1 (22,60), we found H3 peptide binding of mUHRF1 not (V1) or only mildly (V2) affected by hemimethylated DNA (data not shown). Based on these findings we concluded that TTD-driven H3K9me3 mark recognition in mUHRF1 variants is differently established compared to hUHRF1.

### Alternative splicing of mLinker 2 blocks mTTD H3K9me3-binding in mUHRF1 V1

**Figure 4.**
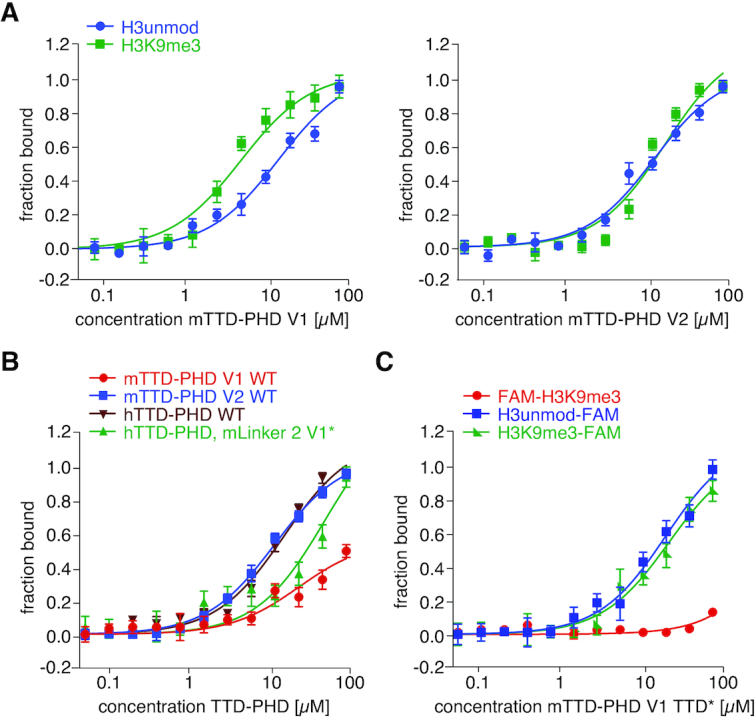
Alternative splicing of mLinker 2 blocks mTTD H3K9me3-binding in mUHRF1 V1. (**A**) Titration series of recombinant mTTD-PHD V1 (left) and mTTD-PHD V2 (right) with H3(1–15)unmodified-FAM or H3(1–15)K9me3-FAM peptides were analysed by fluorescence polarization. Data are plotted as average of three independent experiments; error bars correspond to s.d. (**B**) Fluorescence polarization binding experiments as in (A) of the indicated recombinant proteins with FAM-H3(1–15)K9me3 peptide. (**C**) Fluorescence polarization binding experiments as in (A) of mTTD-PHD V1 TTD* with H3(1–15)unmodified-FAM, H3(1–15)K9me3-FAM or FAM-H3(1–15)K9me3 peptides.

Because the two mUHRF1 variants only differ from each other in the insertion of nine amino acids in mLinker 2 (Figure 1A), this must be the cause of their different H3-tail binding behaviour. To determine the impact of mLinker 2 on the interaction of mTTD and mPHD with H3 peptides, we set up quantitative FP measurements using C- or N-terminally fluorophore (FAM)-labelled unmodified/K9-trimethylated H3 peptides. While a C-terminally-labelled H3 peptide (H3unmod/K9me3-FAM) allows binding of both the PHD and TTD (depending on the K9 tri-methylation status), an N-terminally-labelled H3K9me3 peptide (FAM-H3K9me3) enables monitoring TTD-dependent binding independent of a contribution of the PHD (7,28). Since mLinker 4 is not involved in regulating the functional state of the mTTD, we focused on the isolated mTTD-PHD module to investigate the different H3K9me3-binding behaviours of mUHRF1 V1 and V2. As expected, mTTD-PHD V1 bound the C-terminally-labelled peptides with preference for H3K9me3 over unmodified (*K*_D_s at 4.9 and 14.3 μM, respectively). In contrast, mTTD-PHD V2 bound both C-terminally labelled peptides with similar affinity (Figure 4A). While mTTD-PHD V2 and hTTD-PHD interacted with the N-teminally-labelled FAM-H3K9me3 peptide with similar strength (*K*_D_s at 12.3 and 16.2 μM, respectively), mTTD-PHD V1 showed only very weak binding (*K*_D_ > 300 μM, Figure 4B). Apparently, the insertion in mLinker 2 V1 is sufficient for blocking isolated mTTD-dependent/ mPHD-independent H3K9me3-binding.

To test this further, we introduced the nine amino acids insertion of mLinker 2 V1 into the hTTD-PHD module (hTTD-PHD, mLinker2 V1*). Compared to wild type hTTD-PHD, the hybrid protein exhibited substantial decrease in FAM-H3K9me3 peptide binding (*K*_D_ > 100 μM, Figure 4B). In contrast, interaction with C-terminally-labelled H3 peptides was not affected (Supplementary Figure S7A, B).

To verify that the functional mTTD is involved in the observed mTTD-PHD V1 peptide binding we analysed a mutant of its aromatic cage (Y184A/Y187A). As expected mTTD-PHD V1 TTD* failed to show stronger binding with H3K9me3-FAM over H3unmod-FAM. Also, the low interaction with the FAM-H3K9me3 peptide that cannot bind the mPHD was fully lost (Figure 4C). Collectively, these findings suggested that the mLinker 2 insertion in mUHRF1 V1 is blocking mPHD-independent recognition of the H3K9me3 peptide by the mTTD but at the same time setting up a conformational state of the mTTD-PHD module that allows synergistic binding to the H3K9me3 peptide.

### mTTD-PHD V1 and V2 modules exhibit different domain arrangements and dynamic behaviours

To gain further insights into the different conformational arrangements of the mTTD-PHD V1 and V2 modules inferred from *in vitro* binding experiments, we set out to perform structural studies using small angle X-ray scattering (SAXS) and NMR spectroscopy. We observed previously that the isolated hTTD-PHD module has intrinsic dynamic motion mediated by the flexible hLinker 2 and its transient interaction with the hTTD surface groove and R-pocket (68). Depending on the capturing of the R296 residue of hLinker 2 in the R-pocket, either a synergistic (R296 bound to R-pocket) or an independent (R296 free) H3 peptide-binding mode has been observed (7,65,68).

**Figure 5.**
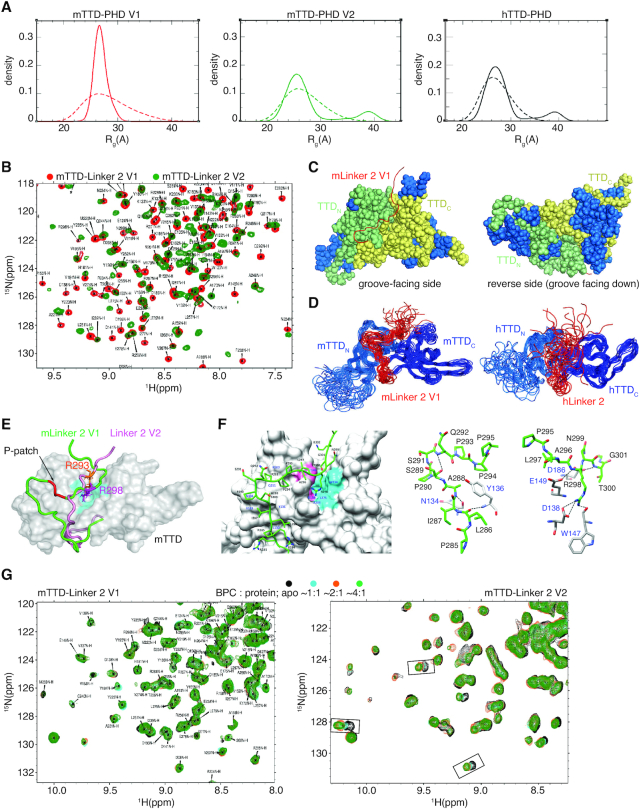
Structural analysis of mTTD-PHD V1 and V2 modules. (**A**) Distribution of *R*_g_ determined by molecular modelling in combination with SAXS profiles of mTTD-PHD V1 (left), mTTD-PHD V2 (middle) and hTTD-PHD (right). Dashed line indicates the initial pool of structures derived from molecular modelling; solid line indicates the SAXS-fitted ensembles of TTD-PHD conformers. (**B**) Overlay of (^1^H–^15^N) HSQC spectra (centre-zoomed) of mTTD-Linker 2 V1 (red) and V2 (green). Resonances were assigned for the V1 protein (85% complete and deposited in the BMRB (ID 30704)). A full spectral overlay is presented in Supplementary Figure S8B. (**C**) Comparison of amide chemical shifts in (^1^H–^15^N) HSQC spectra of mTTD-Linker 2 V1 and mTTD-Linker 2 V2. Overlapping resonances (coloured blue) are mapped onto the mTTD-Linker 2 V1 structure. These are localized outside of the linker-groove interface, and TTD_N_–TTD_C_ junction. (**D**) NMR structural ensembles of mTTD-Linker 2 V1 (left, PDBID: 6VEE) and hTTD-Linker 2 (right, PDBID: 6VED) showing the relative position of the Linker 2 regions with respect to the TTD. (**E**) NMR-derived structure of mTTD-Linker 2 V1 with surface representation of mTTD in grey and mLinker 2 V1 in green. R298 (magenta – stick representation) of mLinker 2 V1 is anchored to the R-pocket (cyan). The P-patch (i.e. P293, P294 and P295) is colored in red. mLinker 2 V2 (pink) is in flux with respect to the mTTD surface; a snapshot representation derived from molecular modeling is shown, with R293 (orange – stick representation) above the R-pocket. (**F**) NMR structure of the interaction between mLinker 2 V1 (green, residues labeled in black) and mTTD (grey, residues labeled in blue). Surface representation with hydrophobic (cyan) and acidic (pink) residues forming the R-pocket occupied by R298 highlighted is shown on the left. Hydrogen bonds (dashed black lines) that stabilize the mLinker 2 V1-mTTD interaction (upstream of the P-patch: N_A288_–O_N134_, N_Y136_–O_L286_, N_Q292_–O_S289_ and downstream of the P-patch: NH2_R298_–OD1_D138_, NH2_R298_–OD2_D138_, N_R298_–OE2_E149_, NH1_R298_–O_W147_, N_T300_–OE2_E186_) are shown in stick representations in the middle and right. (**G**) (^1^H–^15^N) HSQC titrations of mTTD-Linker 2 V1 (left) and V2 (right) with unlabelled 4-benzylpiperidine-1-carboximidamide (BPC) at various molar ratios.

Considering the major differences in sequence and length of Linker 2 in the different murine and human UHRF1 proteins, we addressed whether this gives rise to different structural and dynamic properties of their respective TTD-PHD modules. We performed SAXS analysis of mTTD-PHD V1 and V2 (Supplementary Figure S7C, D, Table S2), and compared their scattering profiles with that of hTTD-PHD (68). We employed an ensemble approach where SAXS curves were fitted against structures derived from molecular modelling to determine a subset of TTD-PHD conformations that were consistent with the experimental data. Several thousand conformers of the modules were generated using molecular dynamics simulations and rigid body modelling to approximate their available conformation space. The SES method (55) was then used to find the ‘optimal’ ensemble of conformers that were consistent with the scattering profiles. In the case of mTTD-PHD V1, the optimal ensemble consisted entirely of compact conformations, where mLinker 2 is putatively bound to the mTTD surface groove (Figure 5A). For mTTD-PHD V2 and hTTD-PHD, in contrast, their respective optimal ensembles gave rise to bimodal *R*_g_ distributions, with evidence of both compact and extended conformations in which the linker is positioned out of the mTTD groove.

(^1^H–^15^N) TROSY NMR spectra of ^15^N-labelled mTTD-PHD V1 and V2 displayed considerable differences in overall peak positions (Supplementary Figure S8A). The dissimilar TROSY spectra could not be attributed to local structural effects resulting from the insertion into mLinker 2 V1 but indicated that there are global differences in mTTD-PHD V1 and V2 conformations. The large size of the mTTD-PHD modules, and their propensity for aggregation at concentrations needed for NMR experiments (∼200 μM) resulted in relatively poor spectral quality and they were not amenable for full resonance assignments. To help rationalize the mTTD-PHD spectra, we prepared ^15^N-labeled mTTD-Linker 2 of V1 and V2 (Figure 5B, Supplementary Figure S8B, C), as well as mPHD (Supplementary Figure S8D, E), which we fully assigned. Comparing spectra of mTTD-PHD, mTTD-Linker 2 and mPHD (Supplementary Figure S8D, E, G, H) two trends were observed: (i) the mPHD peak profiles changed little in mTTD-PHD versus mPHD and (ii) mTTD-Linker 2 peak profiles changed little within the context of mTTD-PHD versus mTTD-Linker 2. These observations indicated that mPHD does not interact with the mTTD or mLinker 2 in the mTTD-PHD modules and that Linker 2 interaction with the mTTD is conserved in both mTTD-Linker 2 and mTTD-PHD context. We observed a similar trend when comparing spectra of hTTD-Linker 2, hPHD and hTTD-PHD (for which backbone assignments were previously determined (68)) (Supplementary Figure S8I). Comparison of ^1^H–^15^N HSQC spectra of mTTD-Linker 2 V1 and V2 showed significant differences with <50% of the peaks at overlapping positions (Figure 5B, Supplementary Figure S8B, C). Resonances that do overlap are exclusively localized outside of the linker-groove interface, and TTD_N_–TTD_C_ junction (Figure 5C). Once again, this indicated that mLinker 2 V1 and V2 exhibit different modes of interaction with the mTTD leading to different structural and dynamic properties of the respective mTTD–PHD modules.

We were able to fully assign the backbone and the side-chain resonances of mTTD-Linker 2 V1, and determine its solution-state structure (PDBID: 6VEE, Figure 5D–F, Supplementary Figure S8L, Table S3). The mTTD structure is very similar to the previously reported NMR-derived and crystal structures of hTTD ((6); PDBID: 2L3R). Key aromatic residues forming the K9me3-binding cage are conserved, as well as the deep acidic R-pocket found in the surface groove at the junction of the two tudor domains (Figure 5F). Interestingly, we observed several long-range NOEs in NOESY spectra between mLinker 2 V1 and mTTD resonances (Supplementary Figure S8J, K). The resulting structural ensemble shows a well-defined interaction between mLinker 2 V1 and the mTTD; specifically, R298 is positioned in the R-pocket (Figure 5D–F). Due to aggregation issues, we were unable to fully assign the resonances of mTTD-Linker 2 V2 and determine its structure. However, as an additional point of comparison, we fully assigned hTTD-Linker 2 and calculated its solution structure (PDBID: 6VED, Figure 5D right panel, Supplementary Figure S8L, Table S3). In contrast to mTTD-Linker 2 V1, we observed very few long-range NOEs between hLinker 2 and hTTD (Supplementary Figure S8J). The structural ensemble displayed a frayed and disordered hLinker 2 in relation to the hTTD surface groove, consistent with earlier studies of hTTD-PHD (68).

To further confirm the differential Linker 2 dynamics in mUHRF1 V1 and V2, we performed HSQC titrations of mTTD-Linker 2 proteins with 4-benzylpiperidine-1-carboximidamide (BPC). BPC was identified in a hUHRF1 compound screen, and was found to interact with the R-pocket (68). The titration data clearly indicated different levels of mTTD-Linker 2 surface groove interactions for V1 and V2. mTTD-Linker 2 V1 resonances showed no chemical shift perturbations (CSPs) upon addition of BPC, confirming that R298 adopts a stable position in the R-pocket which prevents compound binding (Figure 5G). In contrast, CSPs were observed due to BPC binding to mTTD-Linker 2 V2 implying that there is a population of conformations where its surface groove and R-pocket are exposed.

The PHDs in mouse and human UHRF1 adopt very similar structures. We determined the solution structure of mPHD (PDBID: 6VFO, Supplementary Figure S8F, Table S3) and compared it with the previously reported NMR-derived structure of apo-hPHD (64). Both mouse and human PHDs are characterized by a compact canonical C-terminal histone binding region that coordinates two zinc ions (Supplementary Figure S8F); the backbone RMSD for hPHD and mPHD over this region is ∼1.3 Å. The PHDs in both organisms also have a flexible N-terminal loop region, called the ‘pre-PHD’, that seems non-essential for histone-binding, and that coordinates a third zinc ion (Supplementary Figure S8F).

Concluding our structural analysis, we have demonstrated that R298 of mLinker 2 V1 binds tightly to the mTTD R-pocket. Moreover, the structure of mTTD-Linker 2 V1 showed that the three prolines (P-patch: P293, P294 and P295) of the mLinker 2 insertion form a kink making additional contacts with the mTTD surface groove (Figure 5E, F). In contrast, mLinker 2 V2 only transiently interacts with the mTTD surface groove, resulting in much greater conformational freedom of its mTTD-PHD module. As with R296 in hUHRF1, the corresponding R293 of mLinker 2 does not stably occupy the R-pocket (Figure 5E).

### The P-patch and R298 in mLinker 2 V1 are essential for blocking TTD-dependent H3K9me3-binding

**Figure 6.**
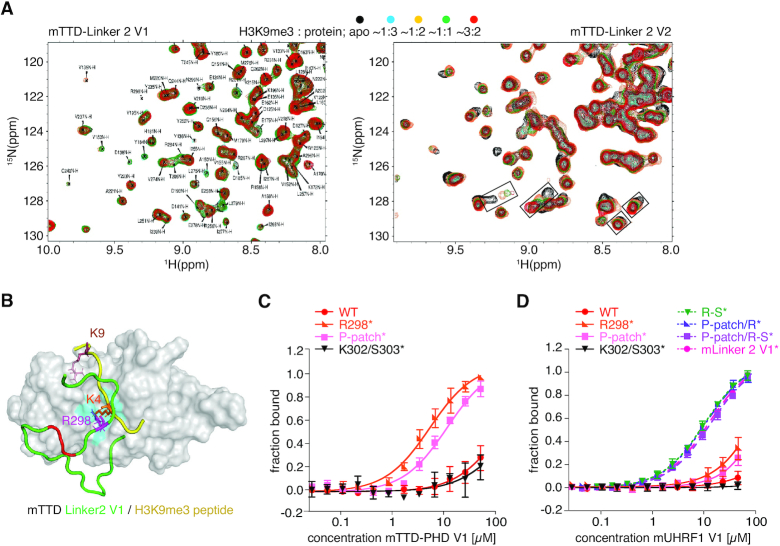
mLinker 2 V1 blocks the surface groove and R-pocket of mTTD. (**A**) (^1^H–^15^N) HSQC titrations of mTTD-Linker 2 V1 (left) and V2 (right) acquired in the presence of unlabeled H3(1–15)K9me3 peptide at different molar ratios. (**B**) NMR-derived structure of mTTD-Linker 2 V1 with surface representation of mTTD in grey and mLinker 2 V1 in green. The P-patch (i.e. P293, P294 and P295) of mLinker 2 V1 is colored in red. The R-pocket (cyan) is occupied by R298 (magenta – stick representation). The model shows the putative positioning of the H3K9me3-tail (yellow) on the surface of the mTTD, as derived from the analysis of the hTTD/H3K9me3 complex ((6); PDBID: 2L3R). The K9me3 residue of the H3-tail would occupy the aromatic cage, and H3K4 the R-pocket. However, because R298 of mLinker 2 V1 (green) is stably bound to the R-pocket and the surface groove is occupied, H3K9me3-binding by the isolated mTTD is blocked in mUHRF1 V1. (**C**) Titration series of recombinant mTTD-PHD V1 WT and R298A (R298*), P293A/P294A/P295A (P-patch*), and K302A/S303A (K302/S303*) mutant proteins with a FAM-H3(1–15)K9me3 peptide were analysed by fluorescence polarization. Data are plotted as average of three independent experiments; error bars correspond to s.d. (**D**) Fluorescence polarization binding experiments as in (C) but using recombinant mUHRF1 V1 WT, R298*, P-patch*, K302/S303* or P293A/P294A/P295A/L297A/R298A (P-patch/R*), R298A/N299A/T300A/G301A/K302A/S303A (R-S*), P293A/P294A/P295A/R298A/N299A/T300A/G301A/K302A/S303A (P-patch/R-S*) and P293A/P294A/P295A/L297A/ R298A/N299A/T300A/G301A/K302A/S303A (mLinker 2 V1*) proteins.

We previously determined that H3 binding to the hTTD in isolation is of composite nature with (i) the hTTD aromatic cage binding to K9me3 and (ii) the hTTD surface groove/R-pocket binding to K4 (6). If the R-pocket is blocked or occupied, hTTD interaction with the H3K9me3 peptide is significantly attenuated (6,28). To confirm that the dynamic behaviour of mLinker 2 V1 and V2 is responsible for differential mTTD H3-binding behaviour, we performed NMR analysis. Titration of mTTD-Linker 2 V1 with a H3K9me3 peptide showed no significant CSPs (Figure 6A). This was in agreement with tight association of mLinker 2 V1 with the surface groove of mTTD and stable positioning of R298 in the R-pocket thereby blocking H3K4 access and eliminating the potential for composite interaction (Figure 6B). However, prominent CSPs were observed when mTTD-Linker 2 V2 was titrated with the H3K9me3 peptide (Figure 6A). The results further confirmed that in this context the mTTD R-pocket is transiently accessible for composite peptide binding.

As predicted from the structural studies, mutation of R298 (R298*: R298A) and the P-patch (P-patch*: P293A/P294A/P295A) resulted in significant gain of binding of mTTD-PHD V1 to the FAM-H3K9me3 peptide compared to the wild type protein as measured by FP (Figure 6C) and suggesting increased accessibility of the mTTD peptide-binding surface groove/R-pocket. In contrast, mutation of the adjacent K302/S303 residues (K302/S303*: K302A/S303A) that are conserved in Linker 2 of mUHRF1 V1, V2 and hUHRF1 (Figure 1A) did not have any effect. When testing the same mutations in the context of full-length mUHRF1 V1, these effects were much less pronounced (Figure 6D). Yet, mutations of additional residues in mUHRF1 V1 Linker 2 enabled FAM-H3K9me3 binding. This was achieved not only by mutation of the complete insertion (mLinker 2 V1*: P293A/P294A/P295A/L297A/R298A/N299A/T300A/G301A/K302A/S303A) but also by some less severe changes, such as R-S* (R298A/N299A/T300A/G301A/K302A/S303A), P-patch/R* (P293A/P294A/P295A/L297A/R298A) and P-patch/R-S* (P293A/P294A/P295A/R298A/N299A/T300A/G301A/K302A/S303A) (Figure 6D). We deduced from these observations that the P-patch and R298 are necessary albeit not sufficient for stably positioning mLinker 2 on the surface of the mTTD and for blocking H3K9me3-binding.

### Linker 2 insertion facilitates synergistic H3K9me3 recognition by the mTTD-PHD module

**Figure 7.**
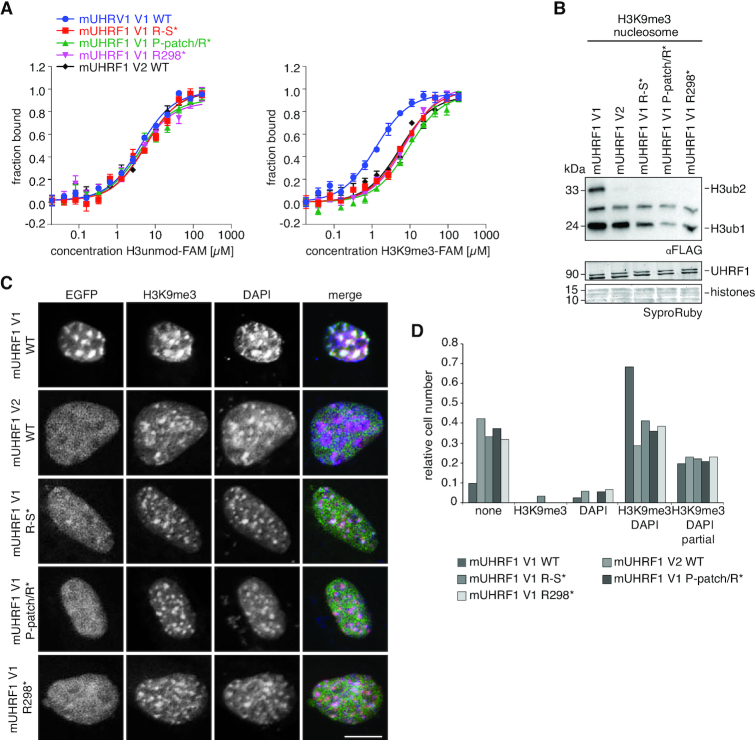
The insertion of mLinker 2 V1 facilitates synergistic H3K9me3-recognition by the mTTD-PHD V1 module. (**A**) Titration series of recombinant mUHRF1 V1 WT, R298A (R298*), P293A/P294A/P295A (P-patch*), P293A/P294A/P295A/L297A/R298A (P-patch/R*), R298A/N299A/T300A/G301A/K302A/S303A (R-S*) and mUHRF1 V2 WT with H3 unmodified- (left) and H3K9me3-FAM (right) peptides were analysed by microscale thermophoresis. Data are plotted as average of three independent experiments; error bars correspond to s.d. (**B**) The recombinant proteins of (A) were subjected to E3 ubiquitin ligase assays using recombinant H3K9me3 mononucleosomes. Reactions were analysed by western blotting (top). Signals for UHRF1 and histone proteins on the stained western blot membrane are shown for loading controls (bottom). Running positions of molecular weight markers (left) and different identified proteins (right) are indicated. (**C**) Representative confocal images of murine NIH-3T3 cells expressing different EGFP-tagged mUHRF1 V1 and V2 WT and mutant proteins (EGFP, green channel). Immunofluorescence staining was performed for H3K9me3 (red channel). DAPI staining marks the DNA (blue channel). Merged images show all three channels simultaneously. Scale bar: 10 μm. (**D**) Co-localization of EGFP-tagged proteins as shown in (C) with H3K9me3 and DAPI-dense regions was assessed visually and is plotted relative to the total number of EGFP-positive cells (*n* > 70).

To determine whether the stable association of mLinker 2 V1 with the mTTD indeed drives the synergistic H3K9me3 peptide-binding mode, we measured interaction of C-terminally FAM-labelled H3 unmodified and H3K9me3 peptides with full-length mUHRF1 V1, V2 and V1 Linker 2 mutants. We predicted that mutation of the Linker 2 insertion that displaces it from the surface of the mTTD would abolish the H3K9me3-binding specificity of mUHRF1 V1. Indeed, R298*, P-patch/R* and R-S* mutations showed weaker binding to the H3K9me3-FAM peptide compared to the WT protein. At the same time, interaction with the H3unmod-FAM peptide was not affected (Figure 7A). Congruent with the reduced binding to the K9me3 H3-tail, the ubiquitylation activity of the mUHRF1 V1 mutant proteins was also decreased to the level of mUHRF1 V2 (Figure 7B). The results supported the notion that the insertion in mLinker 2 V1 directs synergy between mTTD and mPHD. Disruption of the mTTD-Linker 2 association uncouples the mTTD from mPHD resulting in independent modes of peptide recognition (as seen in mUHRF1 V2).

Finally, we asked if the disruption of synergy between mTTD and mPHD by mLinker 2 V1 mutation is sufficient to affect cellular targeting of mUHRF1 V1 to H3K9me3. We transiently overexpressed EGFP-mUHRF1 V1 WT, R298*, P-patch/R* and R-S*, as well as EGFP-mUHRF1 V2 WT in C127 and NIH-3T3 cells. Using confocal microscopy, we found that in both cell lines all three mutations in mLinker 2 V1 caused decreased localization of mUHRF1 V1 to H3K9me3 foci, mimicking the localization of mUHRF1 V2 (Figure 7C, D, Supplementary Figure S9–S11). We concluded that synergistic histone binding by mTTD and mPHD, which is established by the mUHRF1 V1 Linker 2 insertion, is necessary to drive the specific subnuclear localization of mUHRF1 V1. Absence of synergy, either through absence of the insertion (mUHRF1 V2) or mutation of R298, caused a more diffuse localization of mUHRF1, which is not focused at H3K9me3. Interestingly and in contrast to P-patch/R* and R-S*, R298* disrupted synergy between mTTD and mPHD, but did not free the mTTD surface groove for H3 binding (Figures 6D and 7A). This indicated that the differences in subnuclear localization are truly caused by synergistic coupling of mTTD and mPHD in mUHRF1 V1 but not V2.

## DISCUSSION

**Figure 8.**
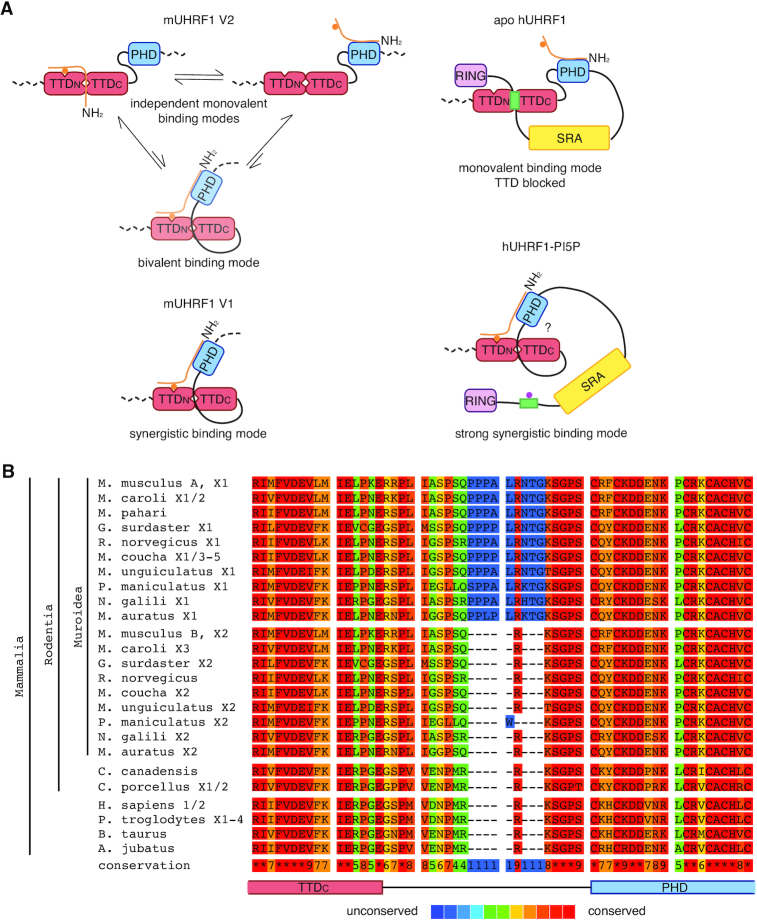
mUHRF1 is functionally and evolutionarily distinct from hUHRF1. (**A**) Model of mUHRF1 and hUHRF1 molecular function. In mUHRF1 V2 the mTTD-PHD module is mostly uncoupled. Neither mLinker 2 V2 nor mLinker 4 is occupying the mTTD surface groove, which is thus available for peptide-binding. The mTTD and mPHD domains bind largely independently to different regions of the H3K9me3 tail. In mUHRF1 V1, the mTTD surface groove is occupied by mLinker 2 V1 and is consequently unavailable for interaction with the H3-tail. This makes the mTTD inefficient in independently binding H3K9me3. Yet, coupling of the mTTD-PHD module facilitates multivalent, synergistic binding of H3K9me3. In apo-hUHRF1, the hTTD surface groove and the hTTD aromatic cage are occupied by the hPBR of hLinker 4. This blocks H3K9me3-binding to this domain and only the hPHD domain is available for H3 N-terminal tail-binding. Allosteric binding of PI5P to the hPBR displaces hLinker 4 from the hTTD. An unknown mechanism causes strong coupling of the hTTD-PHD module that is reflected in very high affinity for the H3K9me3 H3-tail. (**B**) Alignment (PRALINE alignment tool, http://zeus.few.vu.nl/programs/pralinewww/) of the C-terminal region of the TTD_C_, Linker 2 and the N-terminal region of the PHD domains of UHRF1 proteins from different mammalian species. Amino acid sequences correspond to the following NCBI accessions: *Mus musculus* UHRF1 isoform A: NP_001104548.1, *Mus musculus* UHRF1 isoform X1: XP_006523862.1, *Mus musculus* UHRF1 isoform B: NP_001104549.1, *Mus musculus* UHRF1 isoform X2: XP_006523863.1, *Mus caroli* UHRF1 isoform X1: XP_021005580.1, *Mus caroli* UHRF1 isoform X2: XP_021005581.1, *Mus caroli* UHRF1 isoform X3: XP_021005582.1, *Mus pahari* UHRF1: XP_021074194.1, *Grammomys surdaster* UHRF1 isoform X1: XP_028619276.1, *Grammomys surdaster* UHRF1 isoform X2: XP_028619278.1, *Rattus norvegicus* UHRF1: NP001008882.1, *Rattus norvegicus* UHRF1 isoform X1: XP_017451892.1, *Mastomys coucha* UHRF1 isoform X1: XP_031204540.1, *Mastomys coucha* UHRF1 isoform X2: XP_031204541.1, *Mastomys coucha* UHRF1 isoform X3: XP_031204542.1, *Mastomys coucha* UHRF1 isoform X4: XP_031204543.1, *Mastomys coucha* UHRF1 isoform X5: XP_031204545.1, *Meriones unguiculatus* UHRF1 isoform X1: XP_021488698.1, *Meriones unguiculatus* UHRF1 isoform X2: XP_021488701.1, *Peromyscus maniculatus* UHRF1 isoform X1: XP_015856310.1, *Peromyscus maniculatus* UHRF1 isoform X2: XP_015856312.1, *Nannospalax galili* UHRF1 isoform X1: XP_008835189.1, *Nannospalax galili* UHRF1 isoform X2: XP_029421185.1, *Mesocricetus auratus* UHRF1 isoform X1: XP_021089362.1, *Mesocricetus auratus* UHRF1 isoform X2: XP_021089363.1, *Castor canadensis* UHRF1: XP_020027972.1, *Cavia porcellus* UHRF1 isoform X1: XP_023418294.1, *Cavia porcellus* UHRF1 isoform X2: XP_023418295.1, *Homo sapiens* UHRF1 isoform 1: NP_001276980.1, *Homo sapiens* UHRF1 isoform 2: NP_037414.3.3, *Pan troglodytes* UHRF1 isoform X1: XP_001139916.2, *Pan troglodytes* UHRF1 isoform X2: XP_016790233.1, *Pan troglodytes* UHRF1 isoform X3: XP_001139745.1, *Pan troglodytes* UHRF1 isoform X4: XP_016790234.1, *Bos taurus* UHRF1: NP_001096568.1, *Acinonyx jubatus* UHRF1: XP_026905369.1. Protein isoforms Xn/ NCBI accessions ‘XP_’ are derived from the genome sequence and have varying levels of transcript or protein homology support. These represent predicted proteins annotated on the NCBI RefSeq contigs.

In this study, we show that interdomain linker regions control the binding of mouse and human UHRF1 to H3-tail modifications, but this regulation is not conserved between species. Regardless of the high similarity of domain sequence and folding, we found striking differences in the H3K9me3-binding behaviour and its regulation in hUHRF1 and mUHRF1. While hUHRF1 is regulated by the hPBR region in hLinker 4 and its ligand PI5P (Figure 3, (28,67)), mUHRF1 occurs as two splicing variants with distinct binding specificities due to differences in mLinker 2 (Figure 1A). Due to an insertion of nine amino acids mLinker 2 V1 folds back onto the mTTD, with R298 occupying the R-pocket and the P-patch tightly associating with the domain's surface groove. We previously showed that the hPBR region of hLinker 4 binds to the hTTD surface groove, with R649 occupying the R-pocket. When the R-pocket in murine or human UHRF1 is stably bound by a Linker, this effectively blocks the isolated interaction of the TTD with the K9me3 H3-tail (Figures 5 and 8A). Because mLinker 2 V1 is rigidly positioned on the surface of the mTTD, it blocks isolated interaction of the domain with H3K9me3 (Figure 6, Supplementary Figure S5A). However, if the N-terminus of the H3-tail is bound to the mPHD domain, the aromatic cage of the mTTD, which lies outside the Linker 2-binding region, is able to contribute to the interaction, establishing a bivalent, synergistic binding mode. mLinker 2 V1 combines two regulatory features: (i) it blocks interaction of the mTTD with H3K9me3 in the absence of mPHD binding to the H3 N-terminus and (ii) it establishes a synergistic binding mode of mTTD and mPHD in case both domains are targeting the same H3-tail (Figure 8A). Functionally, this is reflected by stimulation of mUHRF1 V1 ubiquitylation activity on H3K9me3-nucleosomes, which is abolished when the synergy between mTTD and mPHD is lost (mutation of mLinker 2 V1). In cells, this results in targeting to H3K9me3 foci, which is dependent on the mTTD domain (Figure 1B-E and Supplementary Figure S4) and especially the synergy between mTTD and mPHD (Figure 7C, D and Supplementary Figure S9). This is illustrated by the delocalization of mUHRF1 V1 from H3K9me3 foci by the R298A mutation that does not open up the mTTD surface groove for H3 binding, but only disrupts synergy between mTTD and mPHD (Figures 6D and 7A). The importance of the R-pocket on the surface of the TTD is further illustrated by its role in recognition of LIG1 and LIG1K126me (8,58).

In contrast, mLinker 2 V2 acts like hLinker 2 adopting different and flexible positions relative to the TTD (7,65,68). Yet, full-length hUHRF1 and mUHRF1 V2 behave differently because, unlike hUHRF1, mUHRF1 is not regulated by the PBR region and PI5P (Figure 3). In mUHRF1 V2 the H3K9me3 N-terminus can compete with the weakly bound Linker 2 allowing for binding of the H3-tail to mTTD without mPHD engagement (Figure 8A). In apo-hUHRF1 the hPBR blocks access of H3K9me3 to the hTTD. In analogy with the synergism between mTTD and mPHD established by mLinker 2 V1, we speculate that in hUHRF1 binding of PI5P to the PBR triggers additional conformational changes that stiffen the hTTD-PHD module thereby enabling the very strong interaction with H3K9me3 observed *in vitro*. In cells hUHRF1 largely localizes similar to mUHRF1 V2 but not V1. This suggests that the synergistic H3K9me3-binding mode induced by PI5P is a regulated event. As mUHRF1 V1 and V2 are differentially expressed, the isolated (mUHRF1 V2, apo-hUHRF1) and constitutive (mUHRF1 V1) or regulated (hUHRF1(PI5P)) synergistic H3K9me3-binding modes of UHRF1 proteins are likely fulfilling different biological functions.

Our data demonstrate the importance of studying full-length proteins and not only isolated domains, whose independent functions may be distinct from their concerted activities. We also highlight the relevance of interdomain linker regions for the complex regulation of multivalent readout by multidomain proteins as exemplified by UHRF1. Interdomain linkers are involved in regulating the functions of structured domains of different proteins in all kingdoms of life. The activity of the bacterial kinase CheA is, for example, regulated by the linker connecting the regulatory and catalytic domains (69). Proteins of the Hsp70 family are allosterically regulated by ATP-binding to the nucleotide-binding domain (NBD); allostery is conferred through induced folding of the linker between the NBD and substrate-binding domain (70).

Comparison of the sequences of Linker 2 and Linker 4 that regulate the binding behaviour of hUHRF1 and mUHRF1 V1 and V2 reveals some common characteristic features. All regulatory linker sequences comprise an essential arginine residue (mLinker 2 V1: R298, mLinker V2: R293, hLinker 2: R296, hLinker 4: R649, Figure 1A, Supplementary Figure S6B) that can be bound by the R-pocket in the TTD surface groove and that is closely accompanied by a threonine or serine residue. In the case of hUHRF1, these sites were shown to be targets of posttranslational modification (65,71), which might affect binding to the TTD surface groove. Such posttranslational modifications and their uncontrolled loss might explain the differences in H3K9me3-binding in cellular hUHRF1 (immunofluorescence) and hUHRF1 from lysate (pull-down) (Figures 1B and 2A). This level of regulation might also be present in the murine proteins. Modulation of the function of interdomain linkers and intrinsically disordered regions by posttranslational modifications or allosteric ligands is a common mechanism for the regulation of protein domain functions. The binding of the chromatin factor BAZ2B to H3K14ac is, for example, enhanced by PARylation of the PHD-BRD linker, which reduces its binding to the PHD and thus allows binding of the H3 N-terminal tail to this domain (72). The activities of protein kinases are generally regulated by posttranslational modifications (phosphorylation) or binding of allosteric ligands to linker regions and intrinsically unstructured domains (reviewed in (73)). To the best of our knowledge, our study demonstrates for the first time that interdomain linker regulation can be mediated by alternative splicing, adding another layer of complexity to the regulatory landscape. Future studies should thus aim at understanding the importance of UHRF1 linker regions and their dynamic regulation by allosteric ligands and posttranslational modifications, as well as differential regulatory mechanisms conferred by sequence variation for UHRF1′s role in DNA maintenance methylation.

Our analyses of mouse *vs*. human UHRF1 and in different cell lines raise possible concerns about data generated in mixed-species experiments. We clearly show that it is imperative for the study of UHRF1 to regard the murine and human proteins as evolutionarily distinct and to be cautious about transferring conclusions drawn from the analysis of mUHRF1 to hUHRF1 and *vice versa*. Multiple sequence alignment and phylogenetic analysis show that the Linker 2 insertion occurred relatively recent in evolutionary terms and is present in the rodent superfamily *Muroidae*, including true mice, rats, hamsters, gerbils and mole rats (Figure 8B). UHRF1 is therefore a prime example for a protein evolving novel functionalities through the alteration of regulatory, intrinsically disordered regions, while core domains remain conserved.

## DATA AVAILABILITY

NMR structures and chemical shift assignments reported in this study have been deposited to the PDB and BMRB under accession numbers 6VED (30703), 6VEE (30704) and 6VFO (30705).

## Supplementary Material

gkaa520_Supplemental_File
