# Supplementary material for: Alternative splicing and allosteric regulation modulate the chromatin binding of UHRF1
Source: Nucleic Acids Res. 2020 Jul 1;48(14):7728–47. doi: 10.1093/nar/gkaa520 (PMC7430637; doi:10.1093/nar/gkaa520)
Supplement: gkaa520_Supplemental_File [file gkaa520_supplemental_file.pdf]

Supplementary Data

Alternative splicing and allosteric regulation modulate the chromatin binding of UHRF1

Maria Tauber[1], Sarah Kreuz[2], Alexander Lemak[3], Papita Mandal[2], Zhadyra Yerkesh[2], Alaguraj Veluchamy[2], Bothayna Al-Gashgari[2], Abrar Aljahani[2], Lorena Cortes Medinia[2], Dulat Azhibek[2], Lixin Fan[4], Michelle S. Ong[5], Shili Duan[3], Scott Houliston[3], Cheryl H. Arrowsmith[3,5] and Wolfgang Fischle[1,2,*]

[1] Laboratory of Chromatin Biochemistry, Max Planck Institute for Biophysical Chemistry, 37077 Göttingen, Germany

[2] Biological and Environmental Science and Engineering Division, King Abdullah University of Science and Technology, Thuwal 23955, Saudi Arabia

[3] Princess Margaret Cancer Centre and Department of Medical Biophysics, University of Toronto, Canada M5G 1L7

[4] Basic Science Program, Frederick National Laboratory for Cancer Research, SAXS Core Facility of the National Cancer Institute, Frederick, MD 21702, USA

[5] Structural Genomics Consortium, University of Toronto, Toronto, Canada M5G 1L7

* To whom correspondence should be addressed. Tel: +966(0)120802498; Email: wolfgang.fischle@kaust.edu.sa

"The authors wish it to be known that, in their opinion, the first four authors should be regarded as joint First Authors".

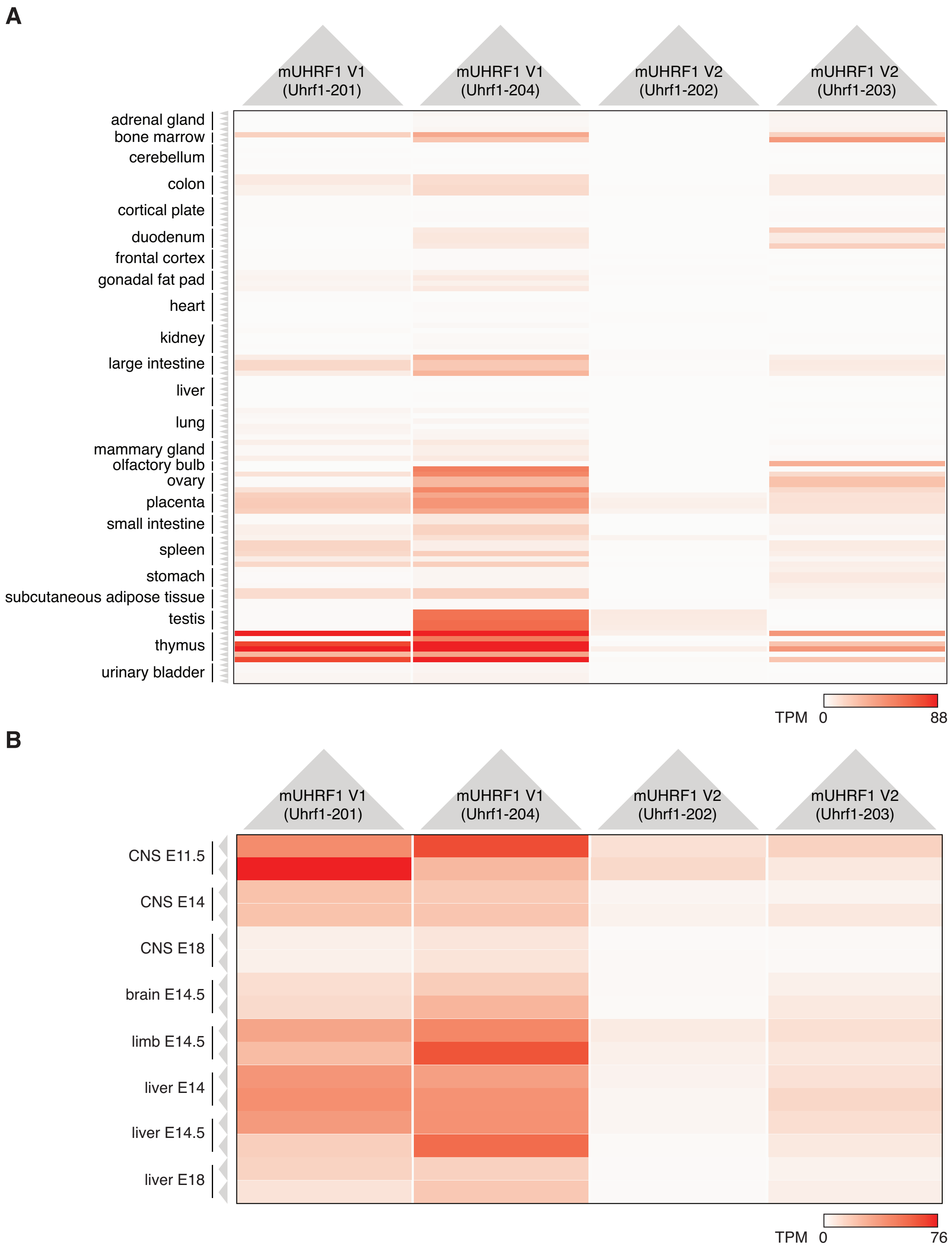

Figure S1: mUHRF1 V1 and V2 are differentially expressed. (**A**) Expression data of mUHR1 mRNAs from 24 types of murine tissues are shown as heatmap. The scale represents transcripts in TPM. (**B**) UHRF1 mRNA quantification for different embryonic developmental stages. Annotation of transcripts according to GencodeM24: transcripts Uhrf1-201 (ENSMUST00000001258.10) and Uhrf1-204 (ENSMUST00000113039.4) encode for mUHRF V1, transcripts Uhrf1-202 (ENSMUST00000113035.3) and Uhrf1-203 (ENSMUST00000113038.3) encode for mUHRF1 V2. Transcripts Uhrf1-201 and Uhrf1-203 contain a different, longer 5'UTR compared to transcripts Uhrf1-202 and Uhrf1-204.

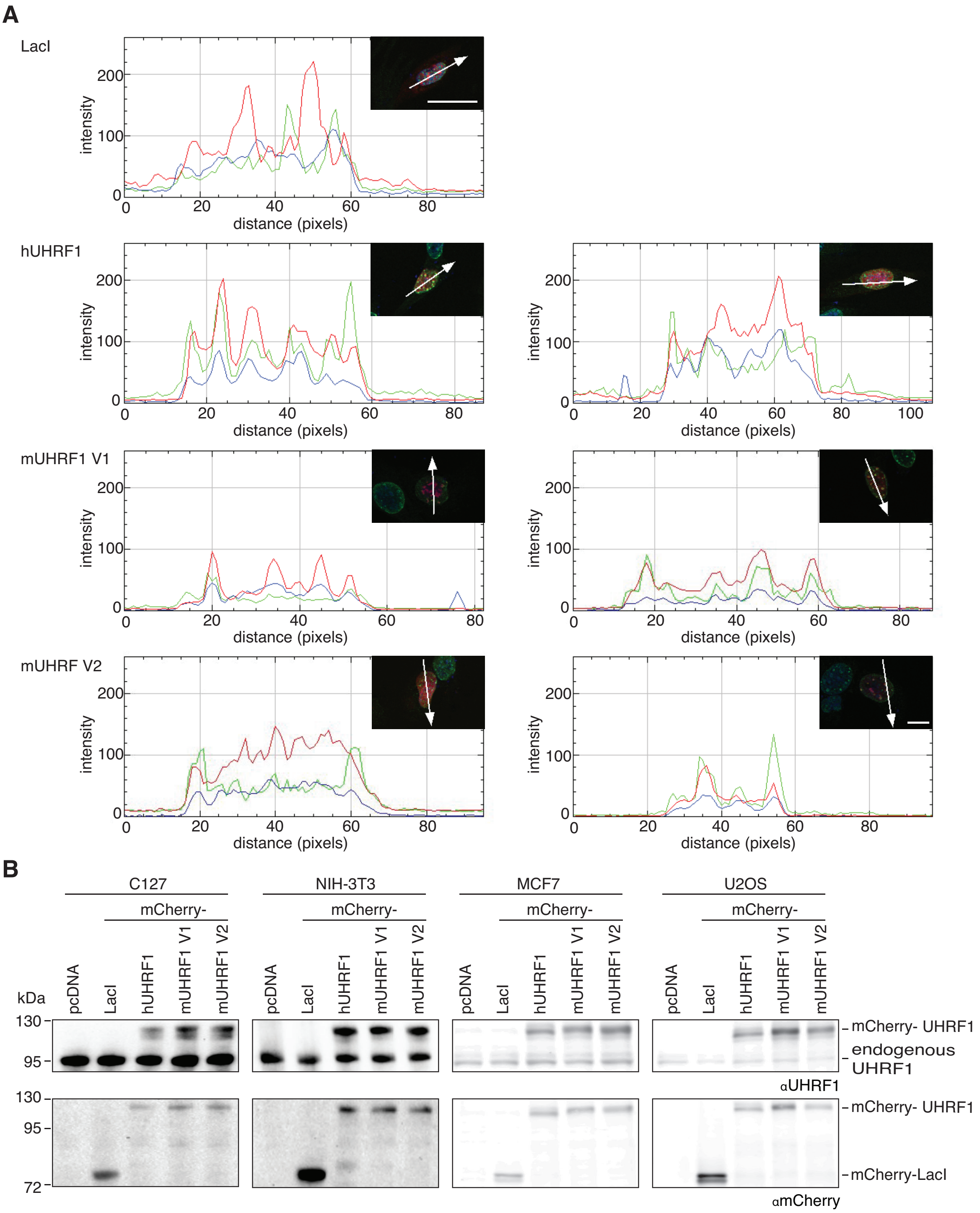

Figure S2: Analysis of different mammalian cell lines transiently expressing mCherry-tagged murine and human UHRF1 proteins. Expression of LacI protein or transfection of carrier plasmid pcDNA3.1 serve as controls. (**A**) Confocal images of representative cells as shown in Figure 1B were analysed using Fiji plotting. Plots represent relative fluorescent signals in mCherry (UHRF1, red), H3K9me3 (green) and DAPI (blue) channels as determined by Adobe Photoshop software. Scale bar: 15 µm (**B**) Western blots of total cell extracts prepared from different cell lines expressing mCherry-tagged mouse and human UHRF1 proteins. Running positions of molecular weight markers (left) and different identified proteins (right) are indicated.

A

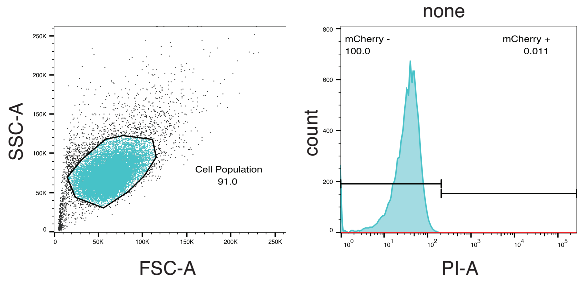

	% of mCherry-positive cells		fluorescence intensity of mCherry-positive cells			
	replicate		median		95-percentile	
	#1	#2	#1	#2	#1	#2
pcDNA (negative control)	0.02	0.01	174	205	573	320
mCherry-LacI	1.56	1.08	433	491	5395	7974
mCherry-mUHRF1 V1	2.53	1.3	468	394	6794	4380
mCherry-mUHRF1 V2	2.42	1.57	413	367	5751	2588
mCherry-hUHRF1	2.55	1.09	473	398	6889	5093

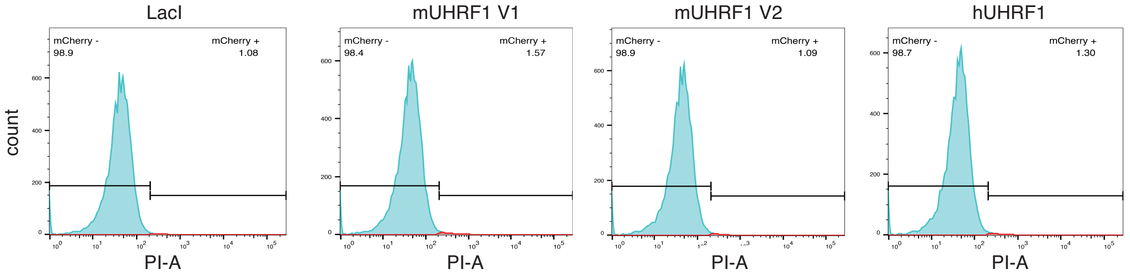

B

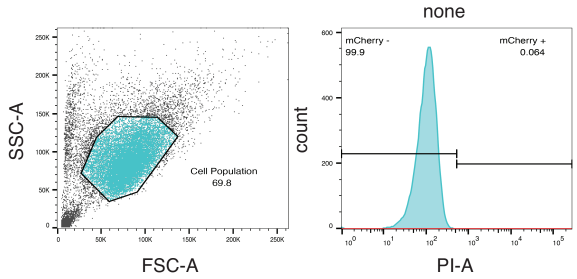

	% of mCherry-positive cells		fluorescence intensity of mCherry-positive cells			
	replicate		median		95-percentile	
	#1	#2	#1	#2	#1	#2
pcDNA (negative control)	0.06	0.03	740	304	7162	1594
mCherry-LacI	22.7	14.8	1466	866	22936	19075
mCherry-mUHRF1 V1	19.9	15.5	1077	846	8368	11351
mCherry-mUHRF1 V2	14.7	9.5	1056	735	6268	7301
mCherry-hUHRF1	16	10.5	1204	779	10655	9513

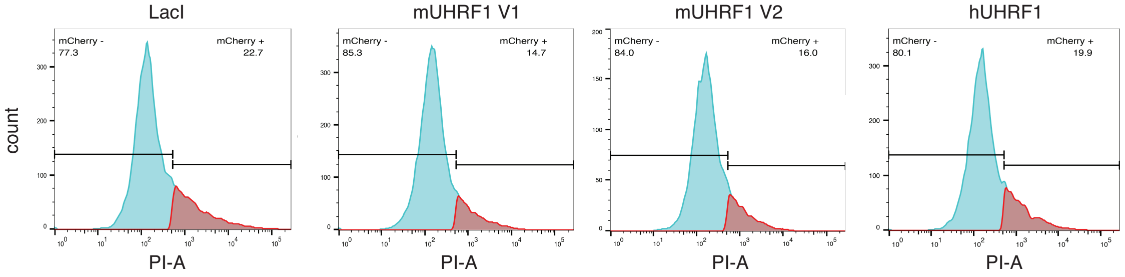

Figure S3: Analysis of different murine cell lines transiently expressing mCherry-tagged murine and human UHRF1 proteins. Flow cytometry analysis of C127 (**A**) and NIH-3T3 cells (**B**) transiently expressing mCherry-tagged murine and human UHRF1 proteins. Expression of LacI protein serves as control. Gating of cell population using forward (FSC) and side scatter (SSC) is shown (upper left). For each condition, mCherry fluorescence intensity per cell (PI-A) is plotted against cell number. Cells transfected with pcDNA vector only (none) were used to set the threshold for the mCherry signal. Percentages of mCherry positive cells, median and 95-percentile of fluorescence intensity of two independent experiments are listed.

A

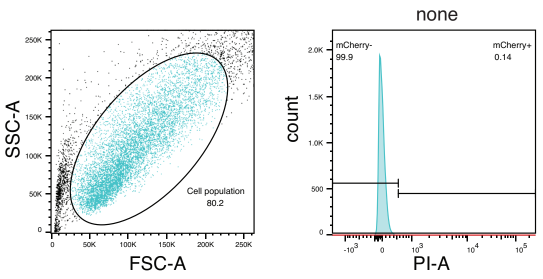

	% of mCherry-positive cells		fluorescence intensity of mCherry-positive cells			
	replicate		median		95-percentile	
	#1	#2	#1	#2	#1	#2
pcDNA (negative control)	0.19	0.14	355	674	2946	1529
mCherry-LacI	0.42	4.37	616	1163	5874	11908
mCherry-mUHRF1 V1	11.7	16.3	614	1190	3083	5103
mCherry-mUHRF1 V2	11.1	17.7	600	1156	2951	4893
mCherry-hUHRF1	12.5	19.4	717	1254	4398	6015

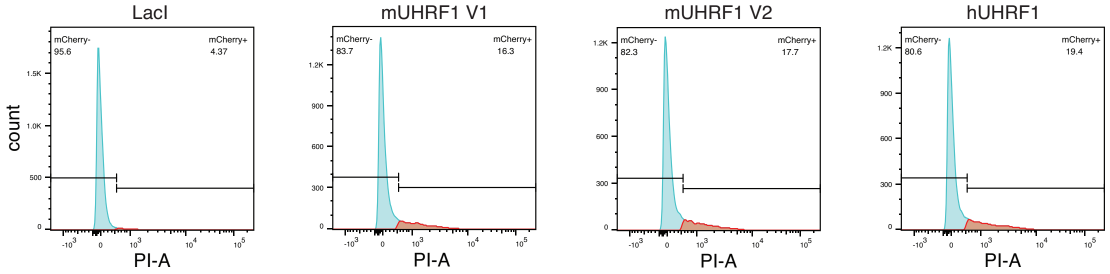

B

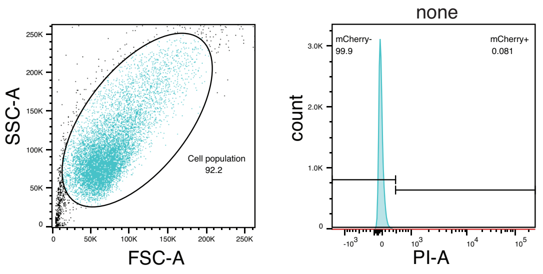

	% of mCherry-positive cells		fluorescence intensity of mCherry-positive cells			
	replicate		median		95-percentile	
	#1	#2	#1	#2	#1	#2
pcDNA (negative control)	0.08	0.08	500	503	25948	657
mCherry-LacI	8.86	23.9	647	1089	5497	14961
mCherry-mUHRF1 V1	43.8	43	895	1392	3754	6067
mCherry-mUHRF1 V2	50.4	43.3	837	1268	3855	5580
mCherry-hUHRF1	46.9	43.9	1008	1564	5812	8448

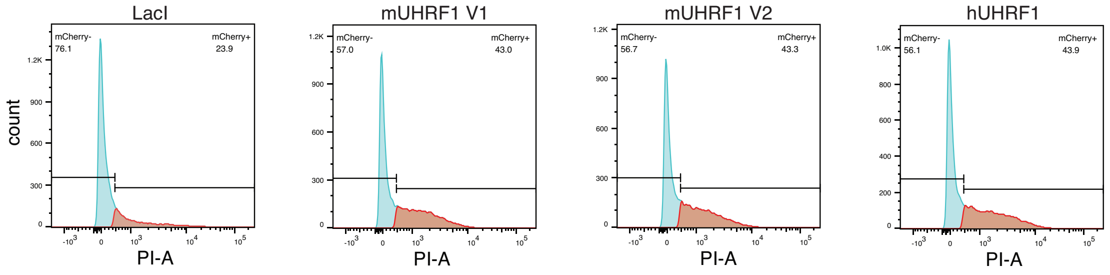

Figure S4: Analysis of different human cell lines transiently expressing mCherry-tagged murine and human UHRF1 proteins. Flow cytometry analysis of MCF7 (**A**) and U2OS cells (**B**) transiently expressing mCherry-tagged mouse and human UHRF1 proteins. Expression of LacI protein serves as control. Gating of cell population using forward (FSC) and side scatter (SSC) is shown (upper left). For each condition, mCherry fluorescence intensity per cell (PI-A) is plotted against cell number. Cells transfected with pcDNA vector only (none) were used to set the threshold for the mCherry signal. Percentages of mCherry positive cells, median and 95-percentile of fluorescence intensity of two independent experiments are listed.

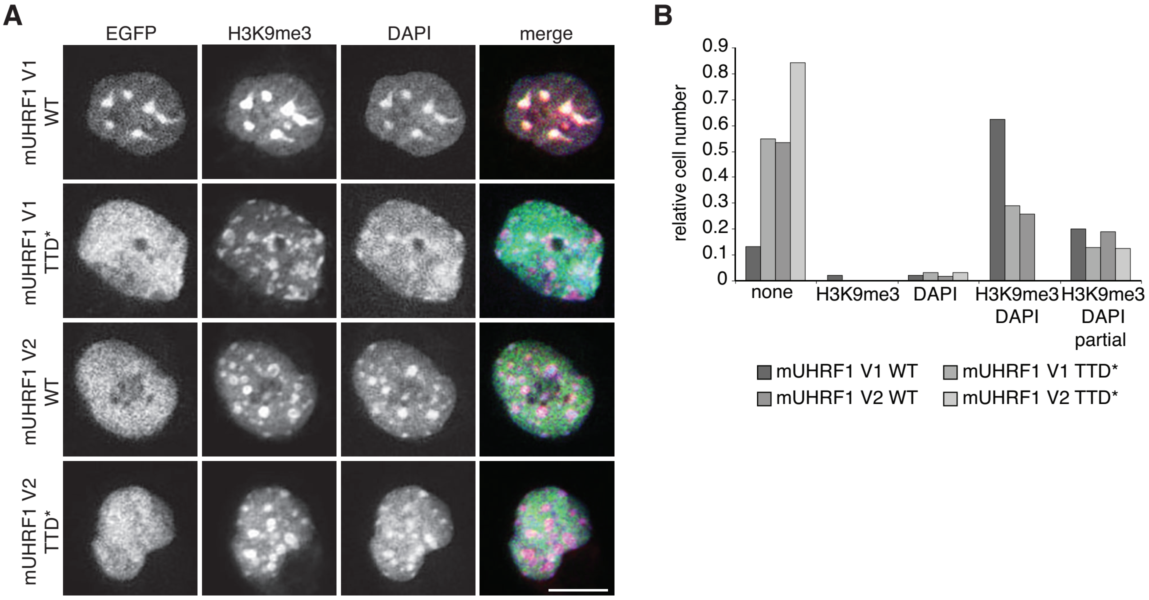

Figure S5: The mTTD domain mediates differences between mUHRF1 V1 and mUHRF1 V2. (**A**) Representative confocal images of murine C127 cells expressing EGFP-mUHRF1 V1 and V2 WT and Y184/Y187A (TTD*) mutant proteins (EGFP, green channel). Immunofluorescence staining was performed for H3K9me3 (red channel). DAPI staining marks the DNA (blue channel). Merged images show all three channels simultaneously. Scale bar: 10 µm. (**B**) Co-localization of EGFP-tagged proteins as shown in (D) with H3K9me3 and DAPI-dense regions was assessed visually and is plotted relative to the total number of EGFP-positive cells (n > 30).

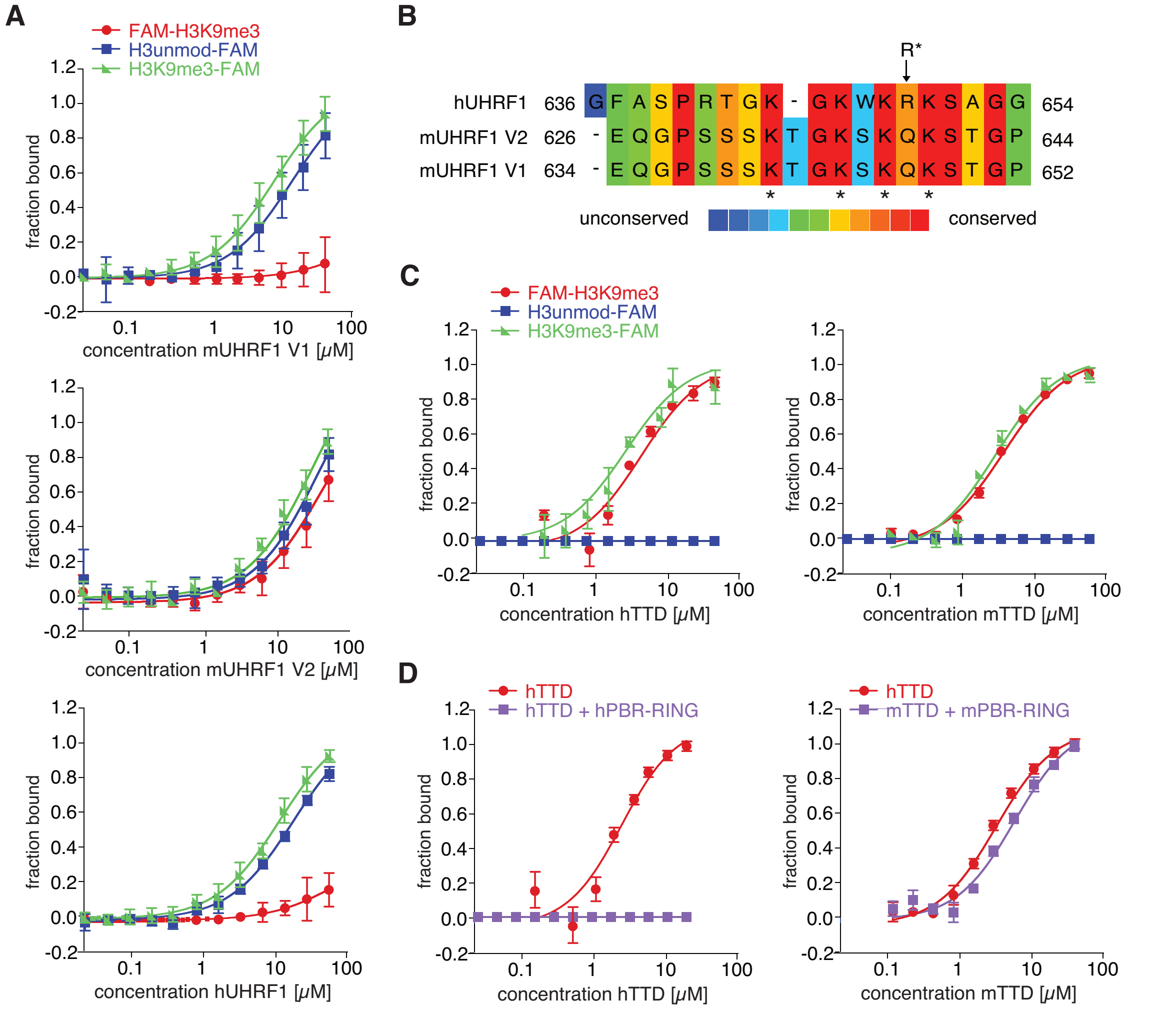

Figure S6: Murine and human UHRF1 proteins differ in their interaction properties with histone tail peptides as well as in their respective PBR regions. (**A**) Titration series of recombinant mUHRF1 V1 (left), mUHRF1 V2 (middle) and hUHRF1 (right) proteins with the indicated H3 peptides were analysed by fluorescence polarization. Data are plotted as average of three independent experiments; error bars correspond to s.d. (B) Fluorescence polarization binding experiments as in (A) analysing the mTTD-PHD V1 module carrying Y184/Y187A (TTD*) mutations. (**C**) Sequence comparison of the PBR region of human and mouse UHRF1 proteins. The arrow marks R649 of the human sequence that mediates a critical interaction with the R-pocket of hTTD. Asterisks mark conserved lysine residues. Hence, the region is referred to as polybasic region (PBR). Alignments were prepared using the PRALINE alignment tool (http://zeus.few.vu.nl /programs/pralinewww/). Amino acid positions correspond to the following NCBI entries: hUHRF1, NP_001276981.1; mUHRF1 V1, NP_001104550.1, mUHRF1 V2, NP_001104548.1. (**D**) Fluorescence polarization binding experiments as in (A) analysing hTTD (left) and mTTD (right). (**D**) Fluorescence polarization binding experiments as in (A) with isolated murine and human TTD domains and FAM-H3K9me3 peptide in absence or presence of 2-fold molar excess of the corresponding Linker 4-RING regions.

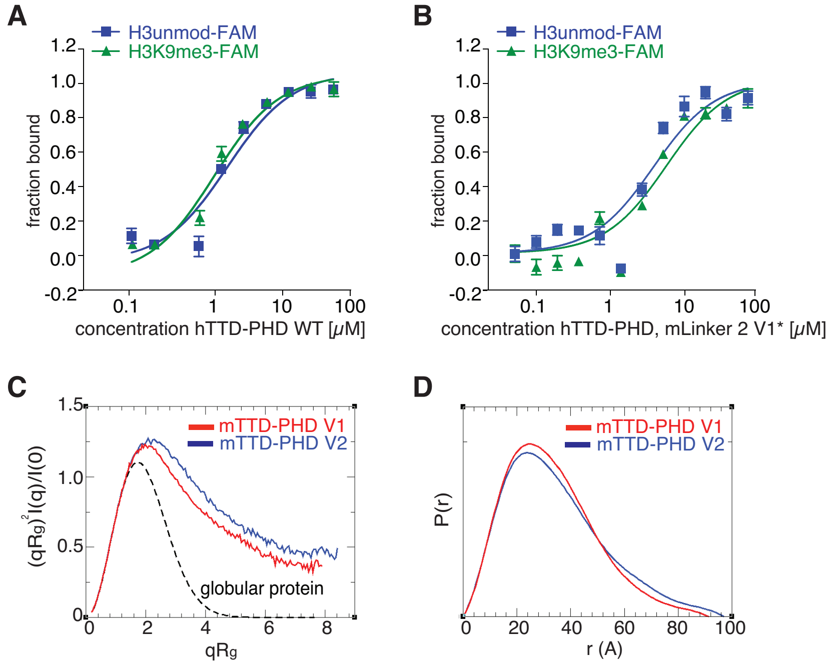

Figure S7: Differences of hTTD-PHD and mTTD-PHD V1 and V2 modules. Titration series of the WT recombinant hTTD-PHD module (**A**) and of the hTTD-PHD module carrying the nine amino acid insertion of mLinker 2 V1 (hTTD-PHD, mLinker 2 V1*, **B**) with the indicated H3 peptides were analysed by fluorescence polarization. Data are plotted as average of three independent experiments; error bars correspond to s.d. (**C**) Comparison of the Rg-based Kratky plots of experimental SAXS curves of mTTD-PHD V1 (red solid line) and V2 (blue solid line) with the theoretical plot for a globular protein of ~31 kDa (black dashed line). (**D**) Normalized pair distance distribution function P(r) of mTTD-PHD V1 (red) and V2 (blue) calculated from experimental data using GNOM (48).

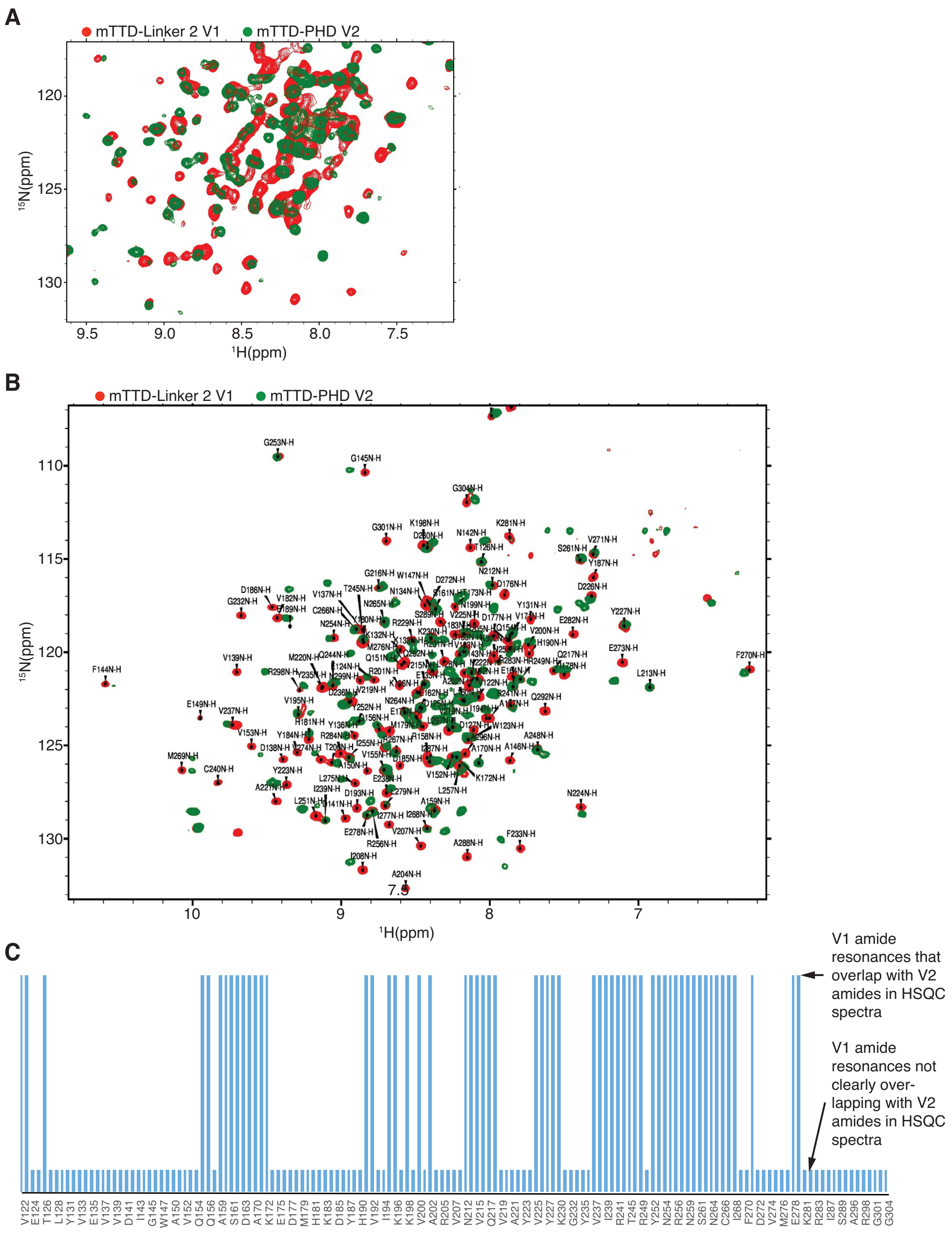

A
mTTD-Linker 2 V1
mTTD-PHD V2
^{15}N(ppm)
^{1}H(ppm)
B
mTTD-Linker 2 V1
mTTD-PHD V2
^{15}N(ppm)
^{1}H(ppm)
C
V1 amide resonances that overlap with V2 amides in HSQC spectra
V1 amide resonances not clearly over-lapping with V2 amides in HSQC spectra
V122 E124 T126 L128 Y131 Y133 E135 V137 V139 D141 I143 G145 W147 A150 V152 Q154 Q156 A159 S161 D163 A170 K172 E175 D177 M179 H181 K183 D185 Y187 H190 V192 I194 K196 K198 V200 A202 R205 V207 N212 V215 Q217 V219 A221 Y223 V225 Y227 K230 G232 Y235 V237 I239 R241 T245 R249 Y252 N254 R256 N259 S261 N264 C266 I268 F270 D272 V274 M276 E278 K281 R283 I287 S289 A296 R298 G301 G304

D

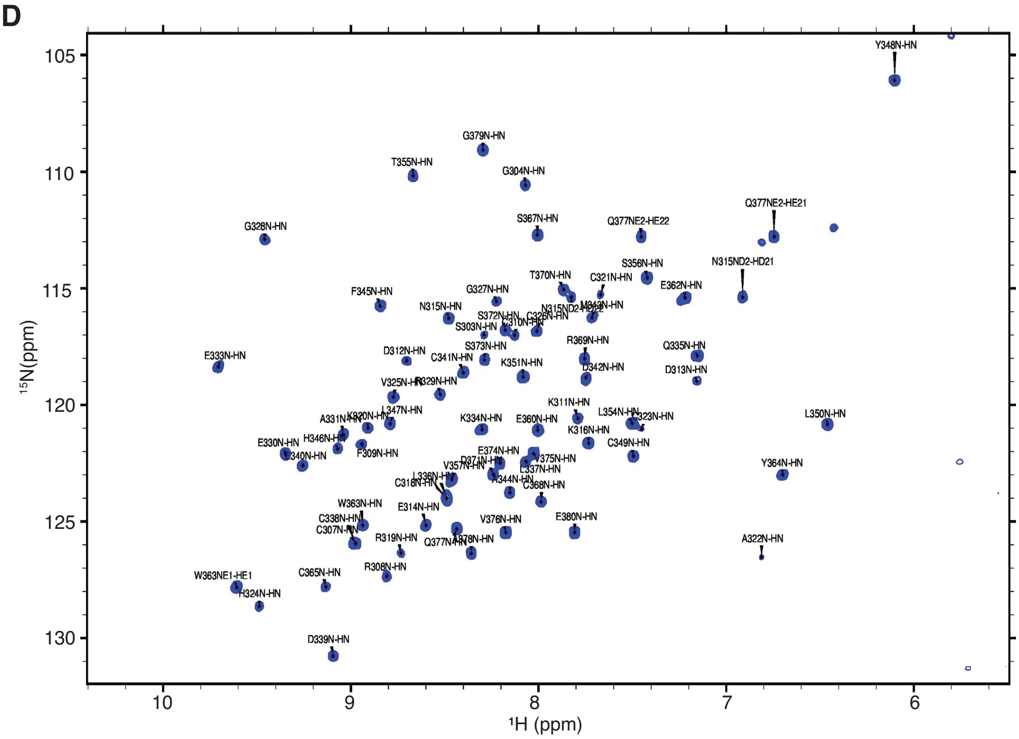
105
110
115
120
125
130
^{15}N(ppm)
10
9
8
7
6
^{1}H (ppm)
Y348N-HN
G379N-HN
T355N-HN
G304N-HN
Q377NE2-HE21
G328N-HN
S367N-HN
Q377NE2-HE22
S356N-HN
N315ND2-HD21
E333N-HN
L350N-HN
Y364N-HN
A322N-HN
W363NE1-HE1
H324N-HN
C365N-HN
D339N-HN

E

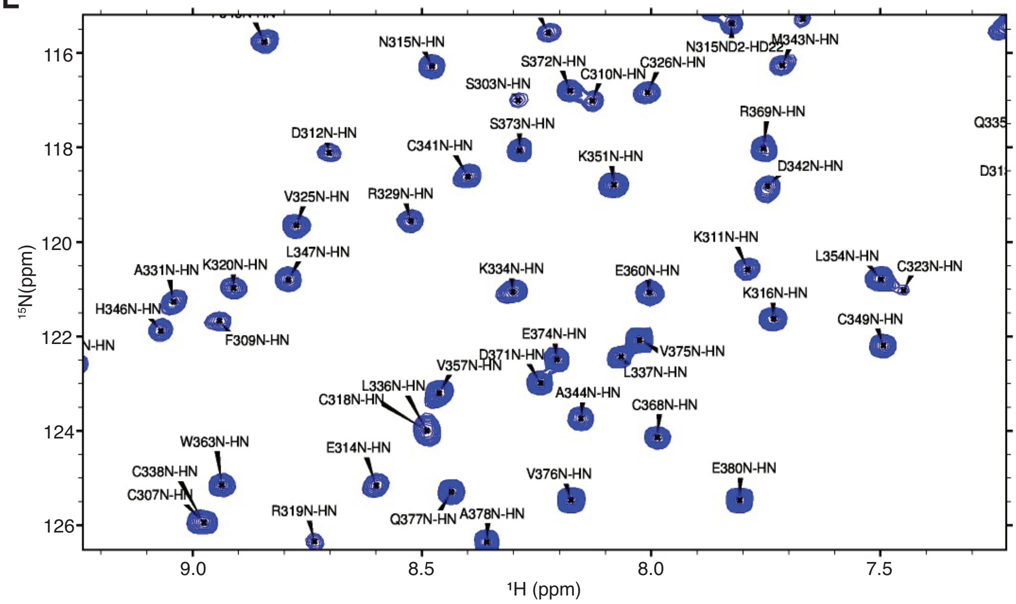
116
118
120
122
124
126
^{15}N(ppm)
9.0
8.5
8.0
7.5
^{1}H (ppm)
N315N-HN
S372N-HN
C310N-HN
C326N-HN
N315ND2-HD22
M343N-HN
S303N-HN
R369N-HN
D312N-HN
S373N-HN
C341N-HN
K351N-HN
D342N-HN
V325N-HN
R329N-HN
K311N-HN
L347N-HN
A331N-HN
K320N-HN
K334N-HN
E360N-HN
L354N-HN
C323N-HN
K316N-HN
H346N-HN
F309N-HN
C349N-HN
E374N-HN
D371N-HN
V375N-HN
V357N-HN
L337N-HN
L336N-HN
C318N-HN
A344N-HN
C368N-HN
W363N-HN
E314N-HN
C338N-HN
C307N-HN
V376N-HN
E380N-HN
R319N-HN
Q377N-HN
A378N-HN

F

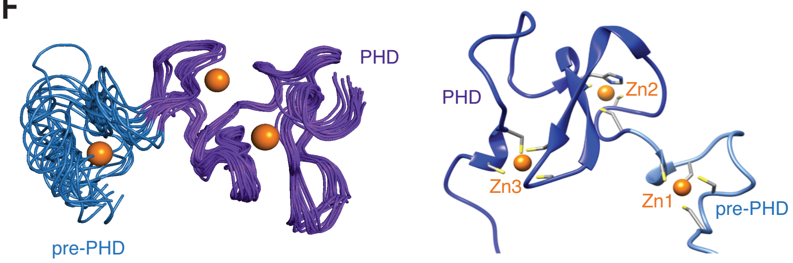
PHD
pre-PHD
PHD
Zn2
Zn3
Zn1
pre-PHD

G

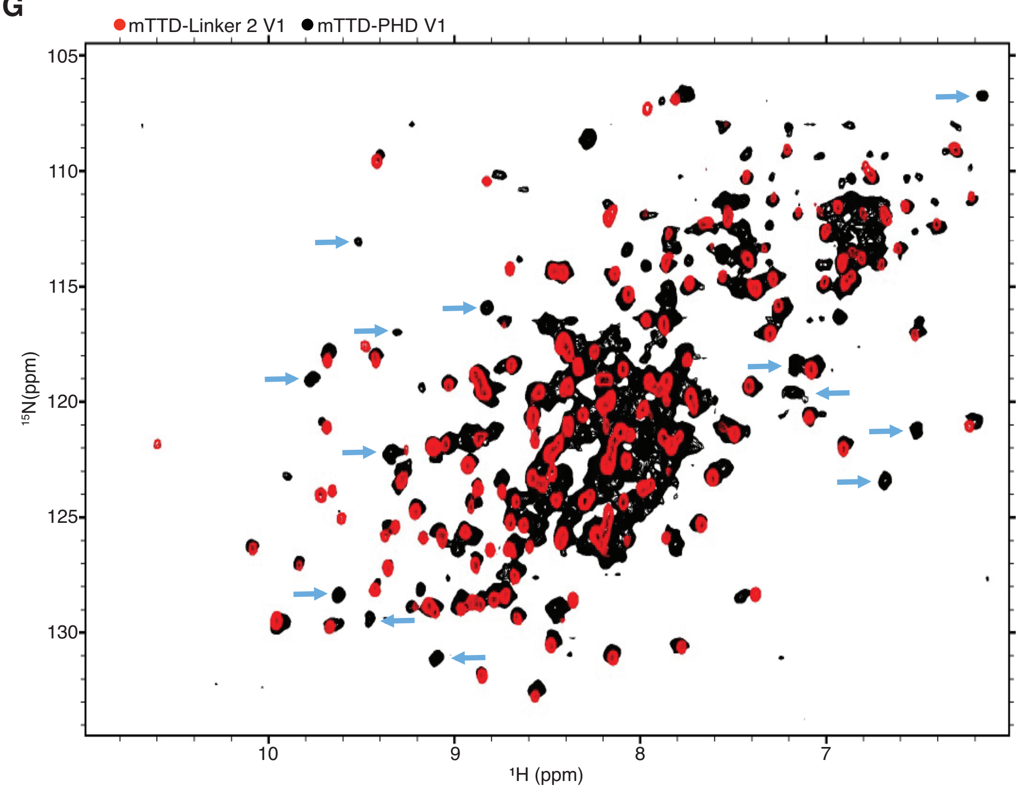
mTTD-Linker 2 V1
mTTD-PHD V1
105
110
115
120
125
130
^{15}N(ppm)
10
9
8
7
^{1}H (ppm)

H

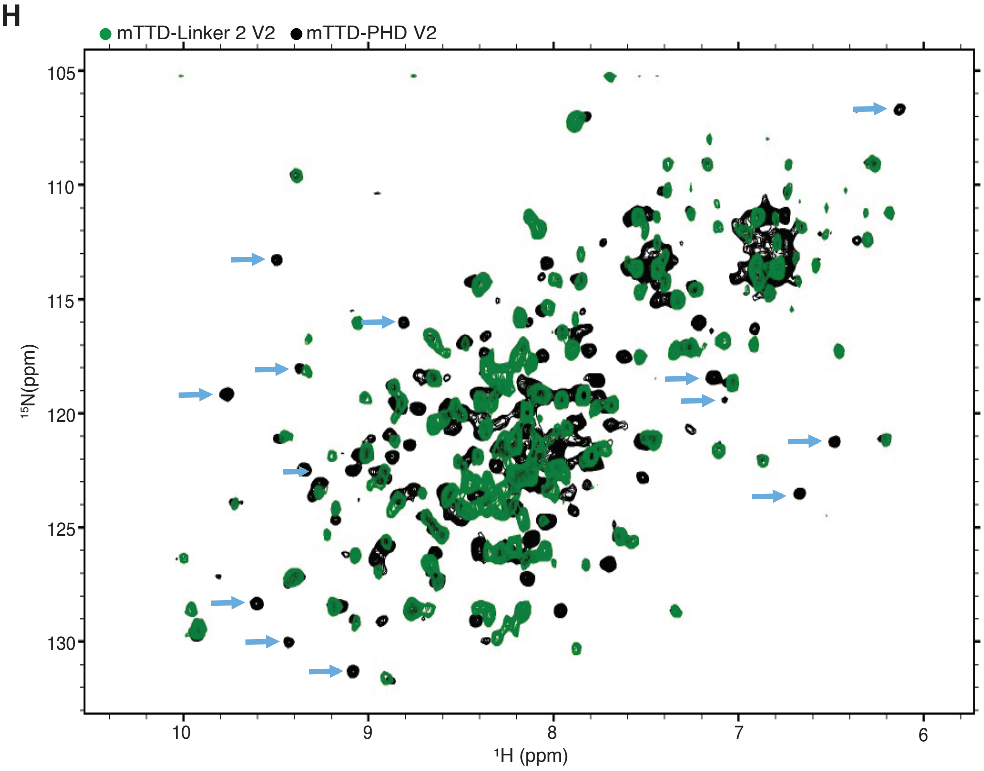
mTTD-Linker 2 V2
mTTD-PHD V2
105
110
115
120
125
130
15N(ppm)
10
9
8
7
6
1H (ppm)

I

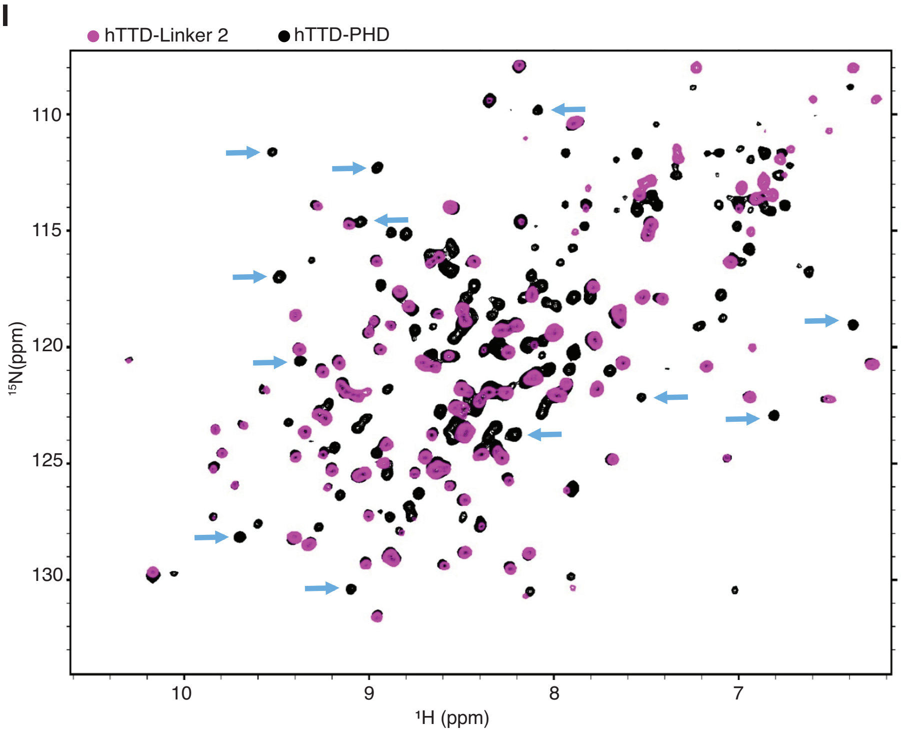
hTTD-Linker 2
hTTD-PHD
110
115
120
125
130
15N(ppm)
10
9
8
7
1H (ppm)

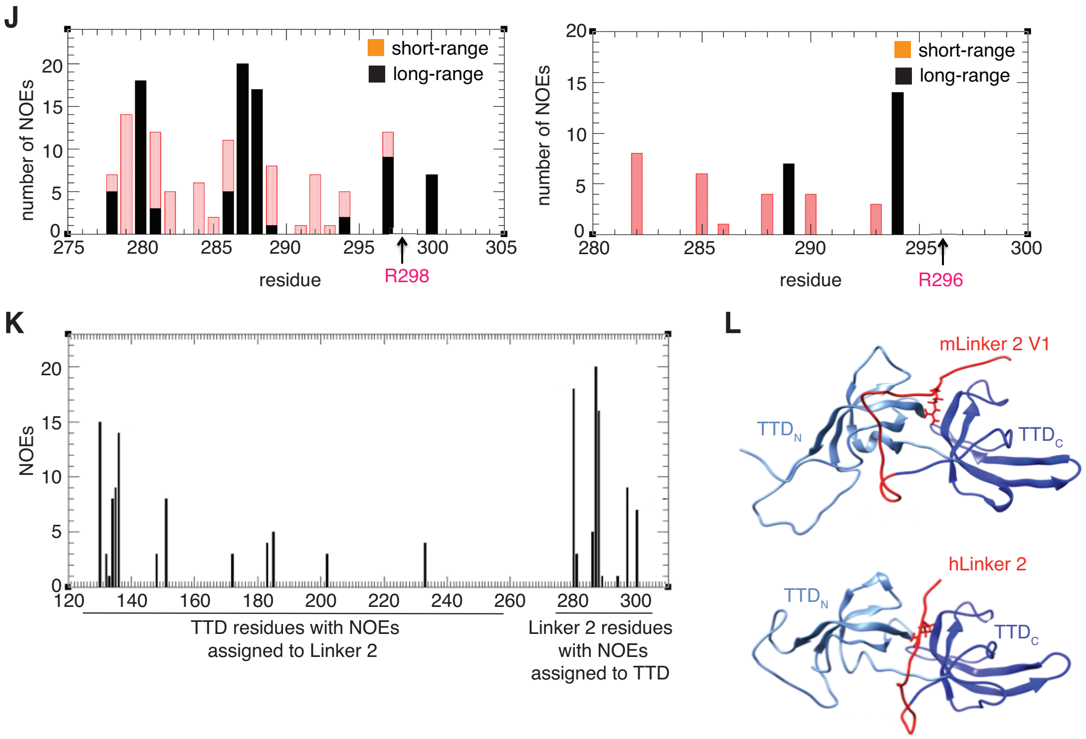

Figure S8: Analysis of different domains and modules of murine and human UHRF1 proteins using NMR. (**A**) Overlay of (^{1}H-^{15}N) TROSY spectra (centre-zoomed) of mTTD-PHD V1 (red) and V2 (green). (**B**) Overlay of full (^{1}H-^{15}N) HSQC spectra of mTTD-Linker 2 V1 (red) and V2 (green). Resonances were assigned for mTTD-Linker 2 V1 (85% complete and deposited in the BMRB, ID 30704). (**C**) Comparison of amide chemical shifts in (^{1}H-^{15}N) HSQC spectra of mTTD-Linker 2 V1 and mTTD-Linker 2 V2 constructs. Bar graph showing the subset of V1 resonances that clearly overlap with those in the V2 construct. (**D**) Full (^{1}H-^{15}N) HSQC spectrum of mPHD with peaks labelled with their resonance assignments. The assignments were deposited in the BMRB (ID 30705) (**E**) Centre-zoomed (^{1}H-^{15}N) HSQC spectrum of the mPHD with peaks labelled with their resonance assignments. (**F**) NMR ensemble of 20 solution structures of mPHD (i.e. mUHRF1$_{303-383}$) is shown by ribbon presentation (left panel). Cartoon representation of the lowest energy structure from the NMR ensemble of mPHD (PDBID: 6VFO) (right panel). Zn ions are shown as orange spheres. (**G**) Overlay of (^{1}H-^{15}N) TROSY spectra of mTTD-PHD V1 (black) and mTTD-Linker 2 V1 (red). Well-resolved putative PHD resonances are marked with blue arrows and were identified based on the comparison with a spectrum of isolated mPHD. (**H**) Overlay of (^{1}H-^{15}N) TROSY spectra of mTTD-PHD V2 (black) and mTTD-Linker 2 V2 (green). Well-resolved putative PHD resonances are marked with blue arrows and were identified based on the comparison with a spectrum of isolated mPHD. (**I**) Overlay of (^{1}H-^{15}N) TROSY spectra of hTTD-PHD (black) and hTTD-Linker 2 (purple). Well-resolved PHD resonances are marked with blue arrows. (**J**) Histograms showing NOEs observed between Linker 2 and TTD resonances (i.e. long-range) in NOESY spectra of mTTD-Linker 2 V1 (top) and hTTD-Linker 2 (bottom). (**K**) For the mTTD-Linker 2 V1 structure calculation, TTD residues with assigned NOEs to Linker 2 resonances, and Linker 2 residues with assigned NOEs to TTD residues are shown. A full list of assigned NOEs used in the structure calculation was deposited to the BMRB (*BMRBID*: 30704). (**L**) Cartoon representations of the lowest energy structures from the NMR ensembles of mTTD-Linker 2 V1 (PDBID: 6VEE, linker shown in red and R298 in stick form, top) and hTTD-Linker 2 (PDBID: 6VED, linker shown in red and R296 in stick form, bottom).

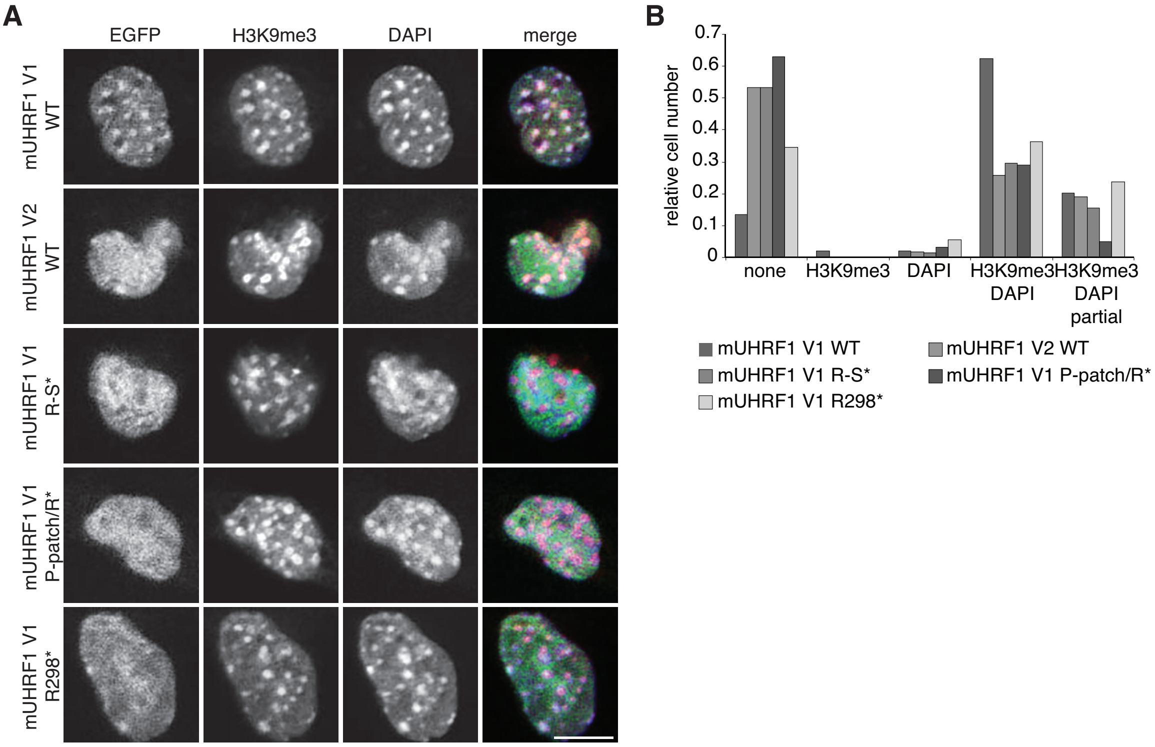

Figure S9: The mTTD domain mediates differences between mUHRF1 V1 and V2. (**A**) Representative confocal images of murine C127 cells expressing different EGFP-tagged mUHRF1 V1/V2 WT and mutant proteins (EGFP, green channel). Immunofluorescence staining was performed for H3K9me3 (red channel). DAPI staining marks the DNA (blue channel). Merged images show all three channels simultaneously. R298*: R298A, P-patch/R*: P293A/P294A/P295A/L297A/R298A and R-S*: R298A/N299A/T300A/G301A/K302A/S303A of mUHRF1V1. Scale bar: 10 µm. (**B**) Co-localization of EGFP-tagged proteins as shown in (A) with H3K9me3 and DAPI-dense regions was assessed visually and is plotted relative to the total number of EGFP-positive cells (n > 30).

A

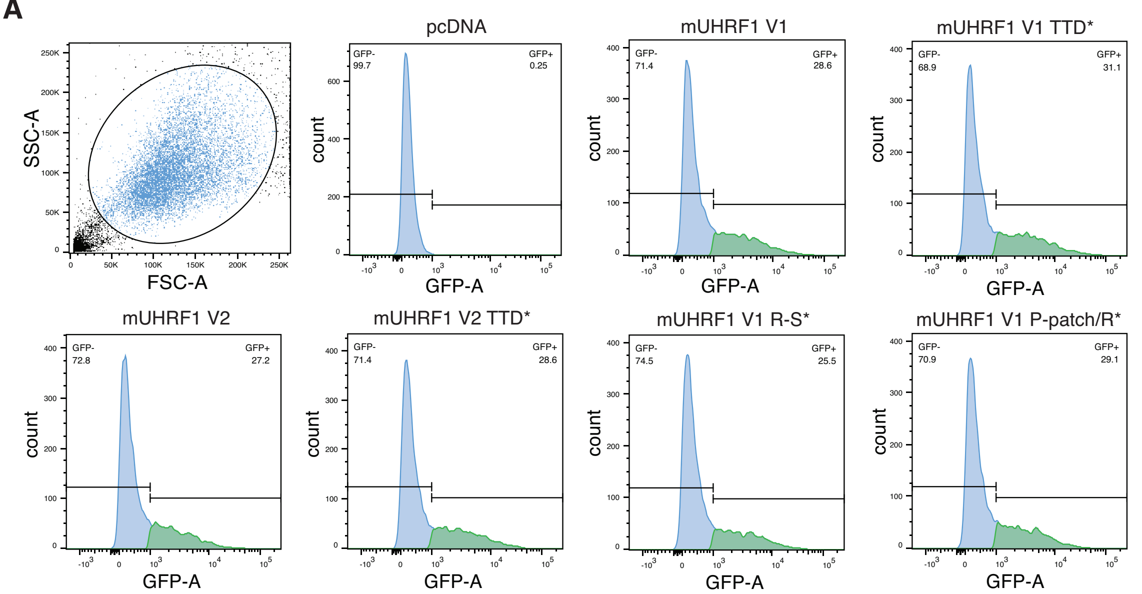

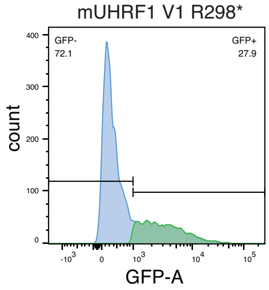

	% of EGFP-positive cells		fluorescence intensity of EGFP-positive cells			
	replicate		median		95-percentile	
	#1	#2	#1	#2	#1	#2
untransfected	0.07	0.1	650	1143	903	3671
pcDNA (negative control)	0.34	0.25	644	1138	946	2438
EGFP-mUHRF1 V1	28.8	28.6	2500	2894	21360	21210
EGFP-mUHRF1 V1 TTD*	36.8	31.1	4380	3189	44548	24919
EGFP-mUHRF1 V2	27.7	27.2	1849	2428	11800	15030
EGFP-mUHRF1 V2 TTD*	26.3	28.6	1884	2975	11881	19392
EGFP-mUHRF1 V1 R-S*	37	25.5	2987	2860	23041	19120
EGFP-mUHRF1 V1 P-patch/R*	44.9	29.1	6516	2804	63821	17613
EGFP-mUHRF1 R298*	35.3	27.9	2644	2922	20911	18115

B

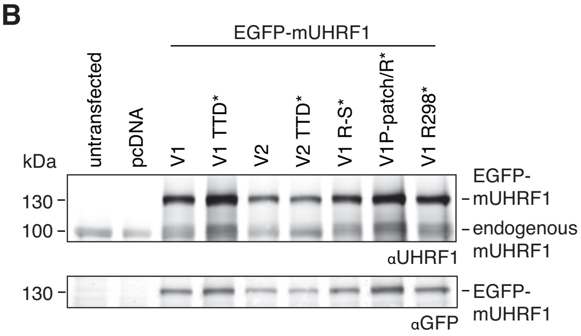

Figure S10: Analysis of NIH-3T3 cells transiently expressing EGFP-tagged mUHRF1 V1 and V2 WT and mutant proteins. (**A**) Gating of cell population using forward (FSC) and side scatter (SSC) in flow cytometry is shown (upper left). For each condition, EGFP fluorescence intensity per cell (GFP-A) is plotted against cell number. Cells transfected with pcDNA vector were used to set the threshold for the EGFP signal. The table lists percentages of EGFP positive cells, median and 95-percentile of fluorescence intensity of two independent experiments. TTD*: Y184/Y187A, R298*: R298A, P-patch/R*: P293A/P294A/P295A/L297A/R298A and R-S*: R298A/N299A/T300A/G301A/K302A/S303A of mUHRF1V1. (**B**) Western blots of total cell extracts prepared from the same cells as analysed in (A). Running positions of molecular weight markers (left) and different identified proteins (right) are indicated.

A

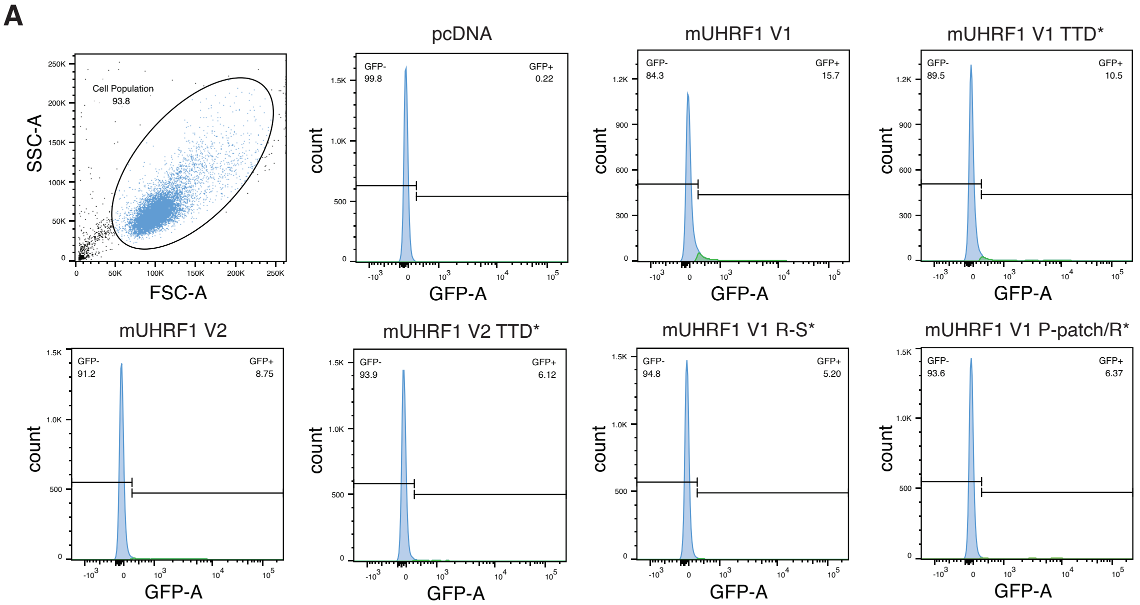

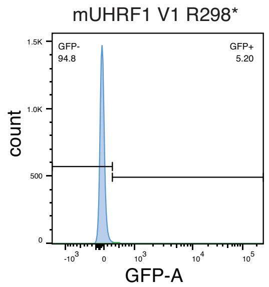

	% of EGFP-positive cells		fluorescence intensity of EGFP-positive cells			
	replicate		median		95-percentile	
	#1	#2	#1	#2	#1	#2
untransfected	0.2	0.19	437	389	628	2140
pcDNA (negative control)	0.26	0.22	475	384	668	1888
EGFP-mUHRF1 V1	3.31	15.7	2350	1084	36724	14383
EGFP-mUHRF1 V1 TTD*	3.73	10.5	1639	1509	26764	17449
EGFP-mUHRF1 V2	3.08	8.75	1576	2193	22132	12438
EGFP-mUHRF1 V2 TTD*	3.98	6.12	3058	1204	18545	7697
EGFP-mUHRF1 V1 R-S*	2.9	5.2	1600	2485	20715	14891
EGFP-mUHRF1 V1 P-patch/R*	3.45	6.37	2744	3107	35251	16497
EGFP-mUHRF1 R298*	2.62	10.5	2400	3372	39478	15100

B

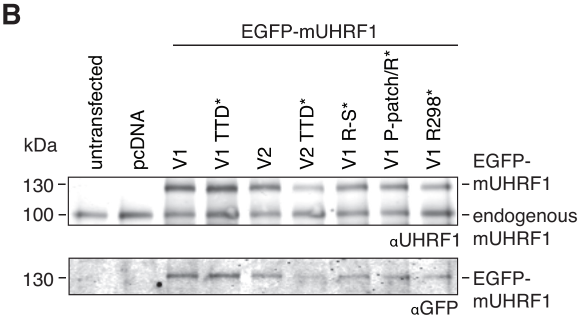

Figure S11. Analysis of C127 cells transiently expressing EGFP-tagged mUHRF1 V1 and V2 WT and mutant proteins. (**A**) Gating of cell population using forward (FSC) and side scatter (SSC) in flow cytometry is shown (upper left). For each condition, EGFP fluorescence intensity per cell (GFP-A) is plotted against cell number. Cells transfected with pcDNA vector were used to set the threshold for the EGFP signal. The table lists percentages of EGFP positive cells, median and 95-percentile of fluorescence intensity of two independent experiments. TTD*: Y184/Y187A, R298*: R298A, P-patch/R*: P293A/P294A/P295A/L297A/R298A and R-S*: R298A/N299A/T300A/G301A/K302A/S303A of mUHRF1V1. (**B**) Western blots of total cell extracts prepared from the same cells as analysed in (A). Running positions of molecular weight markers (left) and different identified proteins (right) are indicated.

Table S1: Dissociation constants (K_D in µM) of interaction of different recombinant WT and mutant murine and human UHRF1 proteins and of corresponding isolated domains and modules with different histone H3-tail peptides as determined by microscale thermophoresis (MST) or fluorescence polarization (FP) measurements. nb, not binding; – not determine; [a] measured by MST; [b] measured by FP.

[a]	H3unmod	H3K9me3	H3R2me2sK9me3
mUHRF1 V1	5.3 +/- 0.2	1.4 +/-0.05	28 +/- 1
mUHRF1 V2	5.8 +/- 0.3	4.5 +/-0.2	14 +/- 1
hUHRF1	2.5 +/- 0.3	1.5 +/-0.1	16 +/- 1
mUHRF1 V1 R-S*	5.1 +/- 0.5	6.2 +/-0.2	-
mUHRF1 V1 P-patch/R*	4.1 +/- 0.3	6.3 +/-0.2	-
mUHRF1 V1 R298*	4.6 +/- 0.3	6.7 +/-0.2	-

[b]	H3unmod-FAM	H3K9me3-FAM	FAM-H3K9me3
mTTD	nb	2.7 +/- 0.3	3.4 +/- 0.2
mTTD + mPBR-RING	-	7.1 +/- 0.4	-
mTTD + mPBR	-	3.3 +/- 0.7	-
hTTD	nb	4.9 +/- 0.6	6.3 +/- 0.7
hTTD + hPBR	-	nb	-
mTTD + mPBR-RING	-	nb	-
mTTD - PHD V1	14.3 +/- 2.5	4.9 +/- 0.9	> 300
mTTD - PHD V2	10.5 +/- 1.8	14.4 +/- 2.4	12.3 +/- 0.8
hTTD - PHD	2.4 +/- 0.4	2.6 +/- 0.3	16.2 +/- 2.5
hTTD - PHD, mLinker 2 V1	4.5 +/- 0.5	6.9 +/- 0.7	> 50
mTTD - PHD V1 P-patch*	-	-	10.2 +/- 0.3
mTTD - PHD V1 R298*	-	-	5.9 +/- 0.5
mTTD - PHD V1 K302/S303*	-	-	nb
mTTD - PHD V1 TTD*	19.5 +/- 3.2	21.4 +/- 3.6	nb
mUHRF1 V1 P-patch*	-	-	nb
mUHRF1 V1 R298*	-	-	nb
mUHRF1 V1 K302/S303*	-	-	nb
mUHRF1 V1 WT	12.7 +/- 3.7	6.4 +/- 0.5	nb
mUHRF1 V2 WT	14.8 +/- 2.7	19.8 +/- 5.4	> 50
hUHRF1 WT	15 +/- 0.8	7.7 +/- 0.7	nb
mUHRF1 V1*	-	-	12.6 +/- 0.5
mUHRF1 V1 P-patch/R-S*	-	-	13.9 +/- 0.8
mUHRF1 V1 P-patch/R*	-	-	16.1 +/- 0.6
mUHRF1 V1 R-S*	-	-	11.1 +/- 0.3

Table S2: Experimental SAXS parameters of mTTD-PHD V1 and V2. [a] Measurements on concentration series were carried out for each protein to remove the scattering contribution due to interparticle interactions and to extrapolate the data to infinite dilution. [b] mTTD-PHD V1 (mUHRF1 $V1_{122\text{-}380}$) with C-terminal 6xHis-tag; sample concentrations were 1.15, 2.3 and 5.5 mg/mL. [c] mTTD-PHD V2 (mUHRF1 $V2_{122\text{-}372}$) with C-terminal 6xHis-tag; sample concentrations were 1.5, 3.0 and 4.7 mg/mL. [d] Intensity at q=0. [e] Radius of gyration calculated using a Guinier fit. [f] Radius of gyration calculated using GNOM. [g] Maximum distance between atoms from GNOM. [h] Molecular weight estimated from SAXS using the volume of correlation; parentheses, molecular weight calculated from the sequence. [i] Molecular weight estimated using SAXS MoW2; parentheses, molecular weight calculated from the sequence.

SAXS parameters[a]	mTTD-PHD V1[b]	mTTD-PHD V2[c]
I(0)[d]	0.016	0.013
Rg[e] (Å)	25.8 ± 0.5	26.4 ± 0.6
Rg (Å) real[e]	25.9 ± 0.2	27.4 ± 0.7
D_{max}[g] (Å)	91	96
Mw[h] (kDa)	28.1 (31.1)	27.3 (30.3)
Mw[i] (kDa)	31.4 (31.1)	30.3 (30.3)

Table S3: NMR restraints, structural statistics and quality scores of NMR-derived structures of hTTD-Linker 2, mTTD-Linker 2 V1 and mPHD. [a] NMR ensembles consist of the 20 lowest energy structures out of 100 calculated. [b] Ordered regions, as reported by the PSVS server for hTTD-Linker 2 are 134-161, 171-285 and 287-294; for mTTD-Linker 2 V1 are 134-157, 178-280, 286-289 and 292-297; for mPHD are 306-310, 313-316, 318-361, 363-369, 374-379. [c,d] Calculated using the PSVS server (*http://psvs.nesg.org/*).

	hTTD-Linker 2	mTTD-Linker 2	mPHD
PDBID	6VED	6VEE	6VFO
BMRBID	30703	30704	30705
NMR distance & dihedral restraints:			
Distance Restraints:			
All	2225	2017	951
Intraresidue	577	625	280
Sequential (|i-j|=1)	648	689	341
Medium range (2 ≤ |i-j| ≤ 4)	231	168	131
Long range (|i-j| > 4)	769	535	229
Hydrogen Bonds	53 x 2	54 x 2	3 x 2
Dihedral angle restraints:			
All	234	258	112
ϕ	117	129	56
ψ	117	129	56
Structure Statistics:			
Number of violations in the NMR ensemble[a]:			
Distance restraints (>0.5 Å)	0	0	0.10 ± 0.30
Dihedral angle restraints (>5°)	0	0.10 ± 0.30	0.15 ± 0.36
r.m.s.d. from experimental restraints:			
Distance (Å)	0.018 ± 0.002	0.019 ± 0.002	0.022 ± 0.005
Dihedral angle (°)	0.745 ± 0.086	0.936 ± 0.088	0.552 ± 0.116
r.m.s.d. from idealized covalent geometry:			
bond (Å)	0.0136 ± 0.0001	0.014 ± 0.0002	0.0141 ± 0.0003
bond angles (°)	0.949 ± 0.017	0.936 ± 0.012	0.950 ± 0.025
impropers (°)	2.15 ± 0.009	2.04 ± 0.11	2.15 ± 0.42
Average r.m.s.d. (Å):			
Ordered regions[b]			
Backbone atoms	1.14 ± 0.23	1.26 ± 0.17	1.49 ± 0.41
All heavy atoms	1.57 ± 0.22	1.77 ± 0.16	2.05 ± 0.42
H3 binding region of PHD construct ($mUHRF1_{323-369}$)			
Backbone atoms	-	-	1.65 ± 0.62
‘pre-PHD’ region of PHD construct ($mUHRF1_{307-321}$)			
Backbone atoms - -	1.65 ± 0.62		
Structural Quality Scores:			
Ramachandran plot (%)[c]:			
Residues in most favored regions	81.1	87.5	81.3
Residues in additional allowed regions	18.9	12.4	18.0
Residues in generously allowed regions	0.0	0.0	0.6
Residues in disallowed regions	0.0	0.0	0.0
Global quality scores[d]	Raw Z-score	Raw Z-score	Raw Z-Score
Procheck (phi-psi)	-0.81 -2.87	-0.60 -2.05	-0.65 -2.24
Procheck (all)	-0.63 -3.73	-0.57 -3.37	-0.46 -2.72
MolProbity clash	6.97 0.33	14.49 -0.96	5.55 0.57
